# The Biosynthesis and Transport of Ophiobolins in *Aspergillus ustus* 094102

**DOI:** 10.3390/ijms23031903

**Published:** 2022-02-08

**Authors:** Jingjing Yan, Jiamin Pang, Jianjia Liang, Wulin Yu, Xuequn Liao, Ayikaimaier Aobulikasimu, Xinrui Yi, Yapeng Yin, Zixin Deng, Kui Hong

**Affiliations:** Key Laboratory of Combinatorial Biosynthesis and Drug Discovery, Ministry of Education and School of Pharmaceutical Sciences, Wuhan University, Wuhan 430071, China; YanJing@whu.edu.cn (J.Y.); pangjiamin@whu.edu.cn (J.P.); 15136122881@163.com (J.L.); wulinyu5@whu.edu.cn (W.Y.); 2019303060040@whu.edu.cn (X.L.); 2019303060015@whu.edu.cn (A.A.); 2019303060065@whu.edu.cn (X.Y.); 2019303060002@whu.edu.cn (Y.Y.); zxdeng@whu.edu.cn (Z.D.)

**Keywords:** ophiobolins, unclustered oxidase, transporter, compartmentalized biosynthesis

## Abstract

Ophiobolins are a group of sesterterpenoids with a 5-8-5 tricyclic skeleton. They exhibit a significant cytotoxicity and present potential medicinal prospects. However, the biosynthesis and transport mechanisms of these valuable compounds have not been fully resolved. Herein, based on a transcriptome analysis, gene inactivation, heterologous expression and feeding experiments, we fully explain the biosynthesis pathway of ophiobolin K in *Aspergillus ustus* 094102, especially proved to be an unclustered oxidase OblC_Au_ that catalyzes dehydrogenation at the site of C16 and C17 of both ophiobolin F and ophiobolin C. We also find that the intermediate ophiobolin C and final product ophiobolin K could be transported into a space between the cell wall and membrane by OblD_Au_ to avoid the inhibiting of cell growth, which is proved by a fluorescence observation of the subcellular localization and cytotoxicity tests. This study completely resolves the biosynthesis mechanism of ophiobolins in strain *A. ustus* 094102. At the same time, it is revealed that the burden of strain growth caused by the excessive accumulation and toxicity of secondary metabolites is closely related to compartmentalized biosynthesis.

## 1. Introduction

Terpenoids, with complicated and diverse structures, are the largest category of natural products. Up to date, more than 175,000 kinds of terpenoid-like and terpenoid-derived compounds have been collected in TeroKit (http://terokit.qmclab.com/, accessed on 1 November 2021) [1]. Sesterterpenoids are a class of rare terpenoids containing five isoprenyl units and are mainly isolated from metazoan, plants and fungi [1]. Although the amount of sesterterpenoids only accounts for less than 1.5% of the total terpenoids, many of these compounds show broad-spectrum biological activities and promising application prospects [1]. The 14/18-membered sesterterpenoids synthesized by terpene synthase LcTPS2 from *Leucosceptrum canum* exhibit significant immunosuppressive activity [2]. Asperterpenols A and B isolated from *Aspergillus* sp. 085242 strongly inhibit acetylcholinesterase [3,4]. Nine new sesterterpenoids synthesized by a silent gene cluster in *Aspergillus ustus* 094102 were identified by gene mining, among which aspergilol A and B showed cytotoxic activities against MCF-7 [5].

Ophiobolins belong to sesterterpenoids with a 5-8-5 tricyclic skeleton. In total, 112 ophiobolins have been discovered so far [6,7,8,9,10,11,12,13,14,15,16,17,18], most of which are isolated from fungi of the genus *Bipolaris* and *Aspergillus* [6]. Ophiobolins show excellent biological activities, especially cytotoxicity. Ophiobolin A exhibits notable activities against CLL and P388 cell lines [19,20]. Ophiobolin O can inhibit the proliferation of MCF-7 and reverse the resistance of MCF-7/ADR to adriamycin [21,22]. The pharmacological mechanism study of 6-epi-ophiobolin G shows that it can be used as a potential estrogen receptor down-regulator to treat breast cancer cells [23].

Though researchers have achieved the total chemical synthesis of (+)-ophiobolin A, (-)-6-epi-ophiobolin N and (+)-6-epi-ophiobolin A, it is severely limited by a high cost and low yield [24,25,26]. The first ophiobolin F (**1**) synthase AcOS was found from *A. clavatus* [27]. Following this, an ophiobolin biosynthesis gene cluster (*obl*) from *A. clavatus*, *Bipolaris maydes* producing ophiobolin A and *Emericella variecolor* (renamed to *Aspergillus stellatus*) and producing ophiobolin K (**2**) were reported (Figure S1) [28]. In the *obl*, chimeric terpene synthase OblA_Ac/Bm/As_ catalyzes the formation of backbone **1**. P450 monooxygenase OblB_Bm__/As_ is involved in the oxidative modification of **1** to form ophiobolin C (**3**); then, 6-epi-ophiobolin C (**4**) and 6-epi-ophiobolin N (**5**) were formed by non-enzymatic transformation from **3**. Moreover, it has been proposed that FAD-dependent oxidoreductase OblC_Ac_ catalyzes the C17-allyl formation of **2** in the side chain of **3** [28].

**Figure 1:**
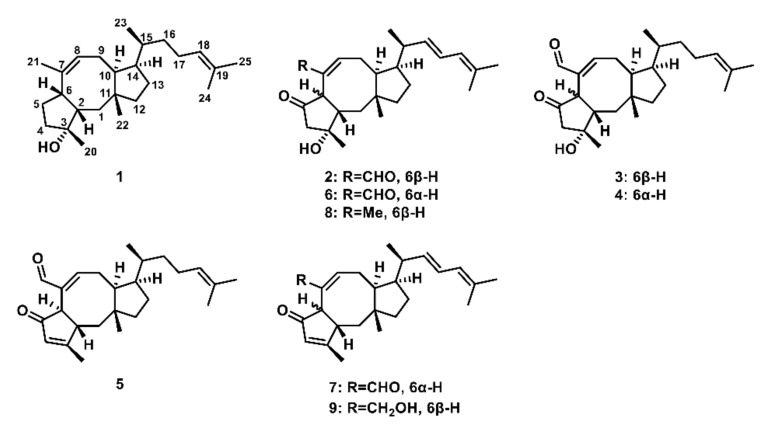
Chemical structure of a part of ophiobolins. Ophiobolin F (**1**); ophiobolin K (**2**); ophiobolin C (**3**); 6-pei-ophiobolin C (**4**); 6-epi-ophiobolin N (**5**); 6-epi-ophiobolin K (**6**); 6-epi-ophiobolin G (**7**); 21,21-O-dihydro-6-epi-ophiobolin G (**8**); 21-deoxyophiobolin K (**9**).

**Figure 2:**
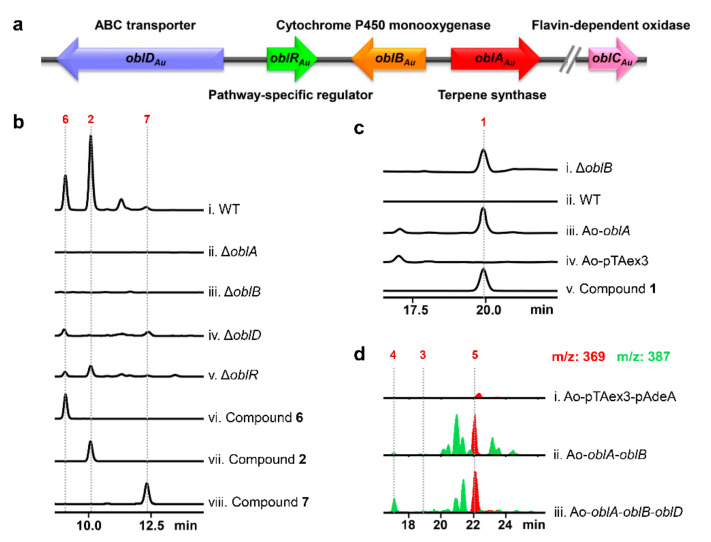
Functional confirmation of genes in ophiobolin biosynthesis gene cluster of *Aspergillus ustus* 094102 (*obl_Au_*). (**a**) Ophiobolin biosynthetic gene cluster in *A. ustus* 094102. (**b**) HPLC profiles of standards and culture extracts from wild-type (WT) strain and mutants at the wavelength of 234 nm. (**c**) HPLC analysis of WT strain, Δ*oblB,* Ao-pTAex3 and Ao-*oblA* at the wavelength of 200 nm. (**d**) LC–ESI-HRMS analysis of culture extracts from *Aspergillus oryzae* transformants. Peaks of m/z 369 (red) and m/z 387 (green) were extracted.

*A. ustus* 094102 produces a series of ophiobolins (**2** and **6**–**9**) (Figure 1) [7,29]. POC8003 (renamed to *obl_Au_*) was verified to be responsible for ophiobolin biosynthesis in *A. ustus* 094102, which contains *au8003* (renamed to *oblA_Au_*), *au8002* (renamed to *oblB_Au_*) and *au8001* (renamed to *oblR_Au_*) (Figure S1 and Figure 2a) [29]. OblA_Au_ has been identified as ophiobolin F synthase [29], and OblB_Au_ was found as a P450 enzyme that could bind to carbon monoxide [30]. However, no gene was found to exist in *obl_Au_* that codes for an enzyme similar to OblC, which may catalyze the formation of a C16=C17 double bond to generate **2**. Additionally, the putative transporter OblD that plays an essential role in the synthesis of ophiobolins [28] has not been clarified in *obl_Au_*.

In this study, we confirm the function of genes in *obl_Au_* and find the unclustered OblC_Au_ that catalyzes the formation of C17-allyl groups. Moreover, the transport process of intermediates and products in ophiobolin biosynthesis is displayed.

## 2. Results

### 2.1. Confirmation of Ophiobolin Biosynthesis Genes in obl_Au_ of A. ustus 094102

In the *obl* of *A. ustus* 094102, both *oblA_Au_* and *oblB_Au_* showed a higher identity of 82.28% and 79.38% with genes in *obl_As_* compared to genes in *obl_Ac_* or *obl_Bm_* by amino acid sequence alignments (Table S1), which showed their similar functions to genes in *obl_As_*. OblR_Au_ was predicted as a Zn(II)_2_Cys_6_ transcription factor with an identity of 21.96%. Moreover, we searched a putative transporter gene *au8000* (renamed to *oblD_Au_*) located upstream of *oblR_Au_*, which showed an identity of 85.64% to *oblD_As_* (Table S1 and Figure 2a).

To confirm the genes involved in ophiobolin biosynthesis in *A. ustus* 094102, we performed RNA-Seq and quantitative real-time (qRT-PCR) analyses at ophiobolin-producing (oph^+^) and non-ophiobolin-producing (oph^−^) conditions (see Supplementary Information). The primers for qRT-PCR are listed in Table S2. According to the calculated log_2_ (fold change), gene expression levels of *oblA_Au_* and *oblB_Au_* upregulated 1795-fold and 760-fold at the oph^+^ condition compared to that of oph^−^, respectively. The genes *oblD_Au_* and *oblR_Au_* also displayed a higher expression level (5.6-fold and 6.2-fold increased, respectively) at the oph^+^ condition compared to those of oph^−^ (Figure S2a). Similarly, the 2^−ΔΔCq^ values also reflected the gene expression upregulation of the four genes at the oph^+^ condition (Figure S2b).

Next, we disrupted these genes separately by using the gene-targeting method based on homologous recombination [31]. Target genes were replaced by the hygromycin resistance gene *hph* and mutants were verified through the use of PCR amplification (Figure S3). The primers are listed in Table S2. The main product, **2**, and its nonenzymatic transformation products, 6-epi-ophiobolin K (**6**) and 6-epi-ophiobolin G (**7**), were identified by LC-MS and NMR analyses and compared with the reported data (Figures S11–13 and S23–28) [7]. In the Δ*oblA_Au_* mutant, all ophiobolins absolutely disappeared compared to the wild-type strain (Figure 2b, i–ii). In the Δ*oblB_Au_* mutant, the yield of ophiobolins was abolished at the UV absorption wavelength of 234 nm (Figure 2b, i and iii), but a compound occurring at the retention time of nearly 20 min and the UV absorption wavelength of 200 nm was detected compared to the wild-type strain (Figure 2c, i–ii). The compound was identified as **1**, confirmed by an NMR analysis and compared with the reported data (Figure S9-10) [27]. This result indicated that **1** was a substrate of OblB_Au_. The production of ophiobolins also decreased significantly in both the Δ*oblD_Au_* and Δ*oblR_Au_* mutants compared to the wild-type strain (Figure 2b, iv–v).

Subsequently, the heterologous expression of genes in *obl_Au_* was performed in *A. oryzae* NSAR1 [27,28,32]. These genes were amplified from the genomic DNA of *A. ustus* 094102 and introduced into *A. oryzae* NSAR1 under the control of an α-amylase promoter. The primers are listed in Table S2. The heterologous expression strains were verified by PCR amplification (Figure S4). Indeed, compound **1** was detected from the mycelium of Ao-*oblA_Au_* (Figure 2c, iii). Two compounds with m/z 387 [M+H] ^+^ and 369 [M+H] ^+^ were detected in Ao-*oblA_Au_*-*oblB_Au_* compared to the strain containing the blank vector Ao-pTAex3-pAdeA (Figure 2d, i–ii and Figure S5, i), which were identified as 6-epi-ophiobolin C (**4**) and 6-epi-ophiobolin N (**5**) by using LC-MS and NMR analyses (Figure S17–22) [33]. Ophiobolin C (**3**) identified by using LC-MS and NMR analyses (Figures S14–16), was accumulated when *oblD_Au_* was introduced into Ao-*oblA_Au_*-*oblB_Au_*, and the yield of **4** and **5** increased significantly (Figure 2d, iii and Figure S5, ii).

### 2.2. Unclustered FAD-Dependent Oxidoreductase OblC_Au_ Is Involved in Ophiobolin Biosynthesis

**Scheme 1:**
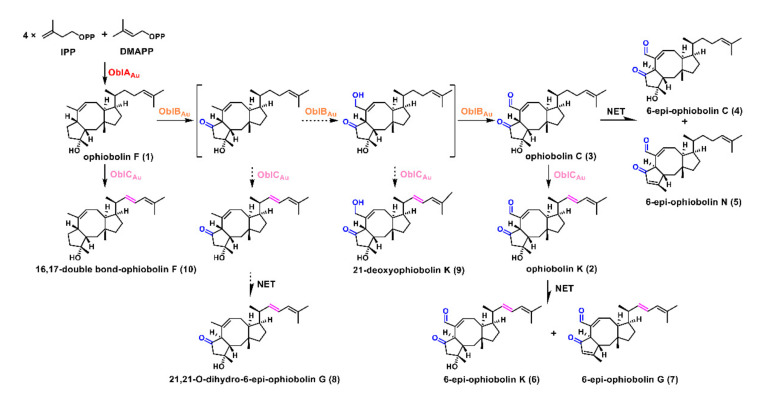
Proposed ophiobolin biosynthetic pathway. NET: non-enzymatic transformation.

According to the above results and the previous report [29], ophiobolin backbone **1** was synthesized by OblA_Au_; multiple oxidations at C5 and C21 of **1** were catalyzed by OblB_Au_ to form **3** (Scheme 1); OblD_Au_ could remarkably improve the ophiobolin production. However, the enzyme responsible for the conversion of **3** to **2** in *A. ustus* 094102 remains unknown.

**Figure 3:**
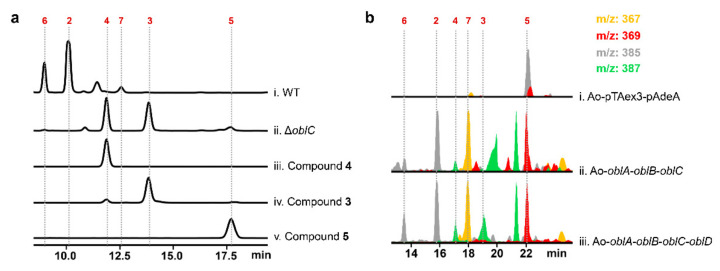
Confirmation of *oblC_Au_* gene function. (**a**) HPLC profiles of standards and culture extracts from wild-type strain and Δ*oblC* mutant at the wavelength of 234 nm. (**b**) LC–ESI-HRMS analysis of culture extracts from *A. oryzae* transformants. Peaks of m/z 367(orange), m/z 369 (red), m/z 385 (gray) and m/z 387 (green) were extracted.

No relative gene was found when we searched genes in the 20 kb upstream and downstream of the *obl_Au_* gene cluster. It was assumed that the gene similar to *oblC_Bm/Ac_* could be located outside of the *obl_Au_* gene cluster in *A. ustus* 094102. Then, we conducted a local blast with the amino acid sequence of *oblC_Ac_*. Three genes coded as *au-orf1*-*3* outside of *obl_Au_* were selected with a 58%, 37% and 33% identity of *oblC_Ac_*. The further detection of the gene expression level of these three genes showed a 161-fold increase in the *au-orf1* gene expression in the oph^+^ condition compared to that of oph^−^ (Figure S2a), while *au-orf2* had no obvious expression differences in two conditions and the expression level of *au-orf3* was downregulated in the oph^+^ compared to the oph^−^ condition (Table S3). Therefore, *au-orf1* (renamed *oblC_Au_*) was chosen as a candidate gene and gene deletion was performed. As expected, ophiobolins **2** and **6**–**7** disappeared, but three new peaks were detected in the Δ*oblC_Au_* mutant compared to the wild-type strain (Figure 3a). These new peaks were confirmed to be ophiobolin **3**–**5** according to the standards.

Then, we introduced *oblC_Au_* into the Ao-*oblA_Au_*-*oblB_Au_* and Ao-*oblA_Au_*-*oblB_Au_*-*oblD_Au_* strains, respectively. In the metabolites of the Ao-*oblA_Au_*-*oblB_Au_*-*oblC_Au_* and Ao-*oblA_Au_*-*oblB_Au_*-*oblC_Au_*-*oblD_Au_* strains, except for the accumulation of products **3**–**5**, one compound with m/z 367 [M+H] ^+^ and two compounds with the same mass of m/z 385 [M+H] ^+^ were detected (Figure 3b and Figure S5, iii-v), which were determined to be **2** and **6**–**7** according to the standards. These results confirmed that **3** was the substrate of OblC_Au_ that was responsible for the dehydrogenation at C16–C17 of **3** to form **2**.

### 2.3. OblC_Au_ also Uses Ophiobolin F as the Substrate

**Figure 4:**
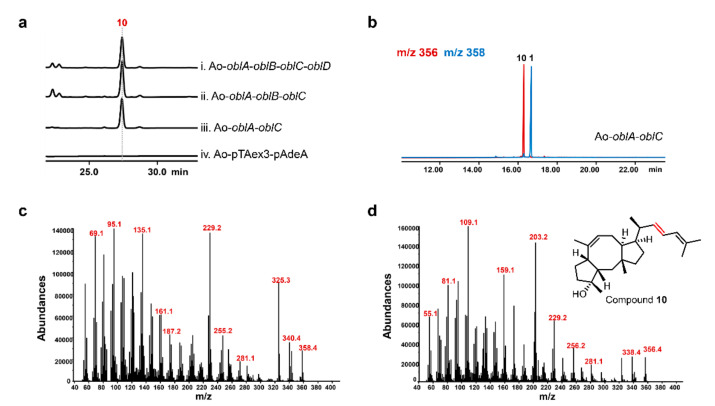
Functional characterization of OblC_Au_. (**a**) HPLC detection of crude extracts of *A. oryzae* expressing *oblA_Au_*-*D_Au_*. (**b**) GC-MS analysis of extracts from Ao-*oblA_Au_*-*oblC_Au_*. (**c**) MS spectra of m/z 358 was extracted. (**d**) MS spectra of m/z 356 was extracted.

Interestingly, another compound with m/z 357 [M+H] ^+^ was detected in Ao-*oblA_Au_*-*oblB_Au_*-*oblC_Au_* and Ao-*oblA_Au_*-*oblB_Au_*-*oblC_Au_*-*oblD_Au_* strains (Figure 4a, i–ii). According to the difference in the molecular weight and MS spectrum of this compound and **1**, we speculated that this compound was a product of dehydrogenation in the side chain of **1** catalyzed by OblC_Au_. To further verify this, we transferred *oblA_Au_* and *oblC_Au_* into *A. oryzae* NSAR1. The HPLC and GC-MS analyses of the metabolites of Ao-*oblA_Au_*-*oblC_Au_* showed **1** and the compound with m/z 357 [M+H] ^+^ were accumulated (Figure 4a, iii and Figure 4b–d). Finally, 45 mg of purified compound was obtained and was identified as 16,17-double bond-ophiobolin F (**10**) by using 1D and 2D-NMR analyses (Figure S29–35 and Table S4–S5). The main differences between **1** and **10** lie in the chemical shifts of the double bond at C16 and C17 (5.21, t, *J* in 9.7; 5.96, m) (Table S4).

Herein, we surmised that **10** could be another substrate besides **1** used for further oxidation by OblB_Au_. Subsequently, compounds **1** and **10** were fed to the strain Δ*oblA_Au_*, respectively. A small amount of **2** and **6** and lots of **10** accumulated when **1** was added, while no product was detected from the strain fed with **10** (Figure S6). The results suggested that OblB_Au_ with the substrate specificity only recognized **1** and not **10** as a substrate. To the opposite, OblC_Au_ could catalyze the C17-allylic formation in the side chain of both **1** and **3** (Scheme 1).

### 2.4. Subcellular Localization Showed the Compartmentalize Biosynthesis of Ophiobolins

**Figure 5:**
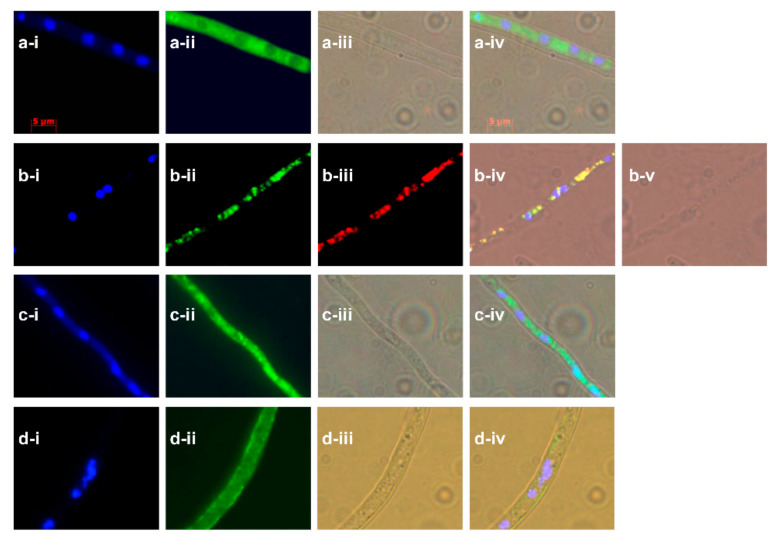
Fluorescence observation of subcellular localization of OblA_Au_, OblB_Au_, OblC_Au_ and OblD_Au_. **a/b/c/d–i**: multiple nuclei localization by DAPI; **a/b/c/d–ii**: eGFP-OblA_Au_, eGFP-OblB_Au_, eGFP-OblC_Au_ and eGFP-OblD_Au_ localization, respectively; **a/c/d–iii** and **b–v**: bright fields. **a-iv**: merge of **a–i**, **a–ii** and **a–iii**; **b–iii**: localization of Mito-Tracker Red CMXRos; **b–iv**: merge of **b–i**, **b–ii**, **b–iii** and **b–iv**; **c–iv**: merge of **c–i**, **c–ii** and **c–iii**; **d–iv**: merge of **d–i**, **d–ii** and **d–iii**.

In order to further determine the function of OblD_Au_, we performed a subcellular localization of proteins involved in ophiobolin biosynthesis. The eGFP tags were fused to the N-termini of OblA_Au_, OblB_Au_, OblC_Au_ and OblD_Au_. Then, the fused genes were introduced into *A. oryzae* NSAR1 under the control of an α-amylase promoter, respectively. The mycelium of strains cultured for 3-5 days was observed by a fluorescence microscope. A microscopic observation of the Ao-*egfp*-*oblA_Au_* strain showed that the green fluorescence signal was widely distributed in the region outside of the nucleus marked in blue by DAPI dye (Figure 5a). It revealed that the OblA_Au_ protein was located in the cytoplasm. The green fluorescence signal in the Ao-*egfp*-*oblB_Au_* strain coincided with the signal of the Mito-Tracker Red CMXRos that specifically marks mitochondria, indicating that the OblB_Au_ protein was mainly present in mitochondria (Figure 5b). OblC_Au_ was also located in the cytoplasm (Figure 5c). The fluorescence signal of OblD_Au_ was observed in the outer membrane of the mycelium (Figure 5d). Based on the 91.3% probability predicted on the online website PSORT II, OblD_Au_ was mainly located in the cell membrane.

To answer why OblD_Au_ was located at the cell membrane, the cytotoxicity of ophiobolins [6] reminded us that the intermediates or final products of strain 094102 may cause toxicity to its growth. Tests of the cytotoxicity of **3**–**5** showed a strong cytotoxic activity against MCF-7 with IC_50_ values of 3.64-4.88 μM, MDA-MB-231 with IC_50_ values of 1.52-2.26 μM and MCF-7/ADR cell lines with IC_50_ values of 0.77-2.71 μM, respectively (Table 1). Based on this, we guessed these compounds could also inhibit the growth of their producing strain. Therefore, we tested the inhibitory effect of intermediate **3** and final product **2** on the growth of *A. oryzae* NSAR1. When the concentrations of **2** and **3** were both 50 μM, the inhibition rates reached approximately 50% (Figure S7). It was obvious that **2** and **3** were toxic to the growth of *A. oryzae*. On the other hand, we noticed that the production of **2** drastically reduced when the Δ*oblD_Au_* gene was disrupted (Figure 2b) and the yield of **3**–**5** obviously increased when the *oblD_Au_* gene was introduced to Ao-*oblA_Au_*-*oblB_Au_* (Figure 2d). It revealed that OblD_Au_ may function as an “antidote” for ophiobolin-producing strains by transporting the toxic compounds outside of the cell membrane.

According to the subcellular localization of the four proteins related to the ophiobolin biosynthesis and “antidote” function of OblD, a workflow was presented (graphical abstract). In the cytoplasm, precursors IPP and DMAPP were elongated and cyclized to ophiobolin skeleton **1** by OblA_Au_; then, **1** was oxidized to **3** by OblB_Au_ in the mitochondria. Compounds **1** and **3** were, subsequently, catalyzed by OblC_Au_ in the cytoplasm to form **10** and **2**, respectively. In order to avoid the toxicity of **2** and **3** in the growth of the strain, these products were transported outside of the cell membrane by OblD_Au_. Since ophiobolins can only be detected from the hyphae, we surmised that ophiobolins exist in the space between the cell wall and cell membrane.

## 3. Discussion

Terpenoid biosynthesis gene clusters usually consist of several genes, encoding the terpene synthase for the skeleton [34] and oxidoreductases for the hydroxylation, aldehydeylation, carboxylation and dehydrogenation [3,4,35]. In the four reported ophiobolin gene clusters, terpenoid synthase, P450 oxidase and flavin-dependent oxidase jointly completed the synthesis of ophiobolins, while the distribution of these genes was irregular. In our study, we found that the flavin-dependent oxidase OblC_Au_ was unclustered. Similarly, in another ophiobolin K-producing strain *A. stellatus*, this enzyme was also not in the *obl_As_*. Additionally, even though OblC_Bm_ was located in the *obl_Bm_* of *B. maydes*, the enzyme responsible for C14-hydroxylation to form ophiobolin A was not found [28]. It is possible that the two enzymes were also unclustered.

Based on the above results, we assumed that OblC_Au_ may have a substrate promiscuity. In *A. ustus* 094102, OblC_Au_ could dehydrogenate intermediates generated in the multi-step oxidation process (Scheme 1), as we discovered 21,21-O-dihydro-6-epi-ophiobolin G (**8**) and 21-deoxyophiobolin K (**9**) from its products [7]. Additionally, the docking results of OblC with **1** and **3** further proved this point. In the catalytic pockets of both models, most residues, especially those near the side chains, were the same (Figure S8), which indicated that the side chain was the core site where OblC recognized substrates, and changes of groups at the rings away from the side chain had little effect on the catalytic activity of OblC. Owing to the substrate promiscuity of OblC_Au_ and the non-enzymatic transformation of natural ophiobolins, nearly 20 ophiobolins were found in the metabolites of *A. ustus* 094102 [7]. FAD-dependent oxidoreductases play an important role in the modification of the terpene skeleton. It has been reported that an unclustered FAD-dependent oxidase EriM responsible for the aldehydation at C-15 in erinacines could catalyze erinacine X, erinacine (14) and erinacine Q to form erinacine ZA, erinacine T and erinacine P, respectively [36]. This discovery suggests that there may be more FAD oxidases with substrate promiscuity responsible for terpenoid biosynthesis, which provides critical clues for the discovery of more terpenoids and combinational synthesis of non-natural products.

The compartmentalized biosynthesis of natural products in fungi is a unique mechanism of higher organisms containing organelles. Compartmentalization can restrict different enzymes and substrates to specific subcellular spaces, thereby effectively preventing non-specific reactions and the cytotoxicity of intermediates or products. The biosynthesis of mycophenolic acid derived from *Penicillium brevicompactum* displays a typical compartmentalization feature, because it relies on the β-oxidation metabolism process in the peroxisome to shorten the isopentenyl long chain specifically [37]. In our research, OblD_Au_ transported ophiobolins outside of the cell membrane to realize the self-resistance of hosts against its toxic metabolites.

Accidentally, we obtained a high-yield ophiobolin C (**3**) strain with 200 mg·L^−1^, which was higher than that previously reported [33]. The cytotoxicity tests showed that all three compounds **3**–**5** had a strong activity against the MCF-7, MDA-MB-231 and MCF-7/ADR cell lines. Additionally, compound **10** exhibited weak activity on MDA-MB-231 (Table 1). Due to **3**–**5** having no stable conjugated groups on the side chain, it is possible to introduce active groups at the end of the side chain through chemical modification [38], which is beneficial to the synthesis of ophiobolin derivatives and provides more valuable lead compounds for cancer therapy.

## 4. Materials and Methods

### 4.1. General Experiential Materials

DNA fragments for construction of plasmids were amplified using I-5^TM^ 2×High-Fidelity Master Mix (TSINGKE Biotech, Beijing, China), whereas PCR for screening of transformants was performed using 2×Es Taq MasterMix (CWBIO, Beijing, China). Primers were synthesized by TSINGKE Biotech (Beijing, China) and sequencing was carried out in TSINGKE Biotech (Beijing, China). The 1 kb ladder (GDSBio, Guangzhou, China) was used as DNA marker. All restriction endonucleases were purchased from New England BioLabs (NEB; Ipswich, MA, USA). T-Vector pMD19 (Simple) was purchased from TaKaRa (Kyoto, Japan). ClonExpress Ultra One Step Clone Kit (Vazyme, Nanjing, China) was used for construction of plasmids rapidly.

High-performance liquid chromatography (HPLC) analysis was performed by LC-20AT instrument (Shimadzu, Kyoto, Japan) with the column of Venusil MP C18 (4.6 × 250 mm, 5μm, Agela Technologies, Delaware, USA). Liquid chromatography coupled with electrospray ionization high-resolution mass spectrometry (LC-ESI-HRMS) on a Thermo Electron LTQ-Orbitrap XL mass spectrometer equipment with an EC-C18 column (4.6 × 150 mm, 2.7μm, Agilent, Santa Clara, CA, USA) was carried out for analysis of ophiobolins. Gas chromatography electron impact-mass spectrometry (GC-MS) analysis conducted by a Varian 450 gas chromatograph (Varian, CA, USA) equipped with a Varian triple quadrupole 320 mass spectrometer (Varian, CA, USA) was carried out for the detection of cultural extract mutants and heterologous expression strains. NMR spectra were collected on a Bruker DPX-400 spectrometer (Bruker BioSpin GmbH, Rheinstetten, Germany).

### 4.2. Strains and Culture Conditions

*Aspergillus ustus* 094102 was isolated from mangrove in our previous work and cultured in potato dextrose agar (PDA) at 28 °C [29]. *Escherichia coli* DH5α (Weidibio, Shanghai, China) was used as the competent cell for construction of all plasmids and cultured in Luria–Bertani (LB) liquid and solid medium at 37 °C. Strain *A. oryzae* NSAR1 (*niaD^−^*, *sC^−^*, ∆*argB*, *adeA*^−^) and plasmids pTAex3-rev-1022 and pAdeA-R were received kindly from professor Ikuro Abe (University of Tokyo). *A. oryzae* NSAR1 was plated on DPY medium (2% dextrin, 1% polypeptone, 0.5% yeast extract, 0.5% KH_2_PO_4_ and 0.05% MgSO_4_∙7H_2_O) at 28 °C.

### 4.3. Software and Databases

Primer premier (Version 5.0) was used for design of primers; SnapGene (Version 2.3.2) was used for plasmid mapping. The analysis and alignment of nucleotide and amino acid sequence were implemented by BioEdit (Version 7.0.9.0). The whole genome sequence of *A. ustus* 094102 was viewed in EditPlus (Version 2.31). ChemDraw 19.0 was used for mapping of compound structure and schemes. The alignment and prediction of amino acid sequence were performed in Basic Local Alignment Search Tool (BLAST, http://blast.ncbi.nlm.nih.gov/Blast.cgi, accessed on 1 November 2021). The subcellular localization prediction of proteins was conducted with online website PSORT II (https://psort.hgc.jp/form2.html, accessed on 1 November 2021).

### 4.4. Genomic DNA Extraction of A. ustus 094102

The method of genomic DNA extraction of *A. ustus* 094102 was described in detail in the previous literature DNA fragments [31].

### 4.5. RNA Extraction of A. ustus 094102 and RNA Sequencing

Ophiobolins were only detected when *A. ustus* 094102 was cultured in solid medium, but not in liquid medium. To analyze the expression level of ophiobolin biosynthesis genes at different conditions, the strain was cultured in liquid fungal II medium (ophiobolin-producing condition) and fungal II medium plates (non-ophiobolin-producing condition) at 28 °C for 3 d, respectively [29]. The mycelia in two conditions were collected and dried, then frozen by liquid nitrogen. The frozen mycelia were used for RNA extraction with TRIzol method [39]. RNA samples were sent to Novogene (Beijing, China) for RNA sequencing. Three biological replicates of each condition were performed for analysis of gene expression level difference.

### 4.6. qRT-PCR Analysis

The RNA samples were reverse transcribed into cDNAs following the manufacture’s protocol of PrimeScript™ RT reagent Kit with gDNA Eraser (TaKaRa, Kyoto, Japan). The cDNAs were analyzed by qRT-PCR with TB Green^®^ Premix Ex Taq™ II (TaKaRa, Kyoto, Japan) on a Bio-Rad CFX96 cycler (Bio-Rad; Hercules, CA, USA). The housekeeping gene *actin* was used for normalization. The primers are listed in Table S2. The relative expression values were calculated using the 2^−ΔΔCq^ method. Cq value represents cycle threshold.

### 4.7. Gene Inactivation in A. ustus 094102

The method for gene inactivation contained the construction of a targeted gene cassette, preparation of protoplast, protoplast transformation and screening of mutants. The detailed protocols were mentioned in the previous paper [31]. The optimized steps for protoplast preparation were added: *A. ustus* 094102 germlings were digested overnight in 10 mL osmotic medium containing 1% driselase from *Basidiomycetes* sp. (Sigma; St Louis, MO, USA) and 2% snailase (Yuanye Bio-Technology Co., Ltd., Shanghai, China). Additionally, the 100-200 μg·mL^−1^ hygromycin B (50 mg·mL^−1^) (Fu Shen, Shanghai, China) was added to stabilized minimal medium for positive transformant screening. The primers for targeting cassette construction and mutants screening were listed in Table S2.

### 4.8. Fermentation and Crude Extracts Analysis of Wild-Type Strain and Mutants

Wild-type strain and mutants were cultured on PDA plates at 28 °C for 10 d. Then, 0.1 g mycelium was scraped from the surface of plates. One milliliter of acetone was added and mixed in a Vortex mixer for 1 min and ultrasound-extracted for 30 min. After centrifugation for 2 min with 12,000 rpm, 10 μL of supernatant was analyzed by HPLC system with 25 min isocratic elution program of CH_3_CN/H_2_O (85:15) and the flow rate of 1 mL·min^−^^1^. The same condition was performed in LC-MS analysis, while the flow rate was reduced to 0.6 mL·min^−^^1^. A 25 min isocratic elution with 100% CH_3_CN was performed for compound **1** analysis by HPLC. For the procedure for GC-MS analysis, refer to the literature [40].

### 4.9. Heterologous Expression in A. oryzae

The heterologous expression in *A. oryzae* included construction of plasmids, protoplast preparation and transformation and fermentation and cultural extracts analysis of *A. oryzae* transformants.

#### 4.9.1. Construction of Plasmids

The nucleotide sequences of *oblA*, *oblB, oblC* and *oblD* were submitted to GenBank with accession numbers OM158715-OM158718, respectively.

Genes *oblA*, *oblB* with its terminator *T_oblB_* (~700 bp), *oblC* with its terminator *T_oblB_* (~700 bp) and *oblD* were amplified from genomic DNA of *A. ustus* 094102 with primers AO-*oblA*-F/R, AO-*oblB*-F and AO-*oblB*-T*_oblB_*-R, AO-*oblC*-F and AO-*oblC*-T*_oblC_*-R and AO-*oblD*-F/R, respectively. A fragment of the *oblA* was inserted into the site of *EcoR*I and *Sma*I in pTAex3-rev-1022 to obtain the plasmid pTAex3-*oblA*. Promoter *amy*1 was amplified from pTAex3-rev-1022 with primers P*amy*-F/R and fused to the 3′ of *oblB-T_oblB_* by overlap PCR. The fused fragment was inserted the linearized pTAex3-*oblA* digested by *EcoR*I to obtain the plasmid pTAex3-*oblA*-*oblB*. Promoter *amy* and terminator *amy* were amplified from pTAex3-rev-1022 with Adea-P*amy*-F/ P*amy*-R’ and T*amy*-F/Adea-T*amy*-R, respectively. Additionally, P*amy*2 and T*amy* were fused to the 5′- and 3′- of *oblD* by using overlap PCR, respectively. The fragment P*amy*2-*oblD*-T*amy* was introduced into the *Spe*I and *Xba*I of pAdeA-R to obtain the plasmid pAdeA-*oblD*. P*amy*3 was amplified with primers P*amy*-*oblC*-F/R and fused into the 5′ of *oblC-T_oblC_*. The fragment P*amy*3-*oblC*-T*_oblC_* was introduced into the *Spe*I and *Xba*I site of pAdeA-R and the *Xba*I site of pAdeA-*oblD* to obtain the plasmids pAdeA-*oblC* and pAdeA-*oblC*-*oblD*, respectively. All primers were listed in Table S2.

#### 4.9.2. Protoplast Preparation and Transformation

The methods for protoplast preparation and transformation were described in the previous paper [5]. The plasmids pTAex3-*oblA* and pTAex3-*oblA*-*oblB* were separately transformed into *A. oryzae* NSAR1 to obtain strains Ao-*oblA* and Ao-*oblA*-*oblB*. Furthermore, adding pAdeA-*oblC*, pAdeA-*oblD* or pAdeA-*oblC*-*oblD* into Ao-*oblA*-*oblB* could obtain the Ao-*oblA*-*oblB*-*oblC*, Ao-*oblA*-*oblB*-*oblD* and Ao-*oblA*-*oblB*-*oblC*-*oblD* strains, respectively. Additionally, Ao-*oblA*-*oblC* was harvested by introducing pTAex3-*oblA* and pAdeA-*oblC* into *A. oryzae* NSAR1. All transformants were verified by PCR amplification.

#### 4.9.3. Fermentation and Cultural Extracts Analysis of *A. oryzae* Transformants

The mycelium of strains was inoculated into 30 g rice medium in 250 mL Erlenmeyer flasks and cultured at 28 °C for 10 d. After that, 100 mL of acetone was added in the cultures and ultrasound-extracted for 30 min. The organic solvent was removed by rotary evaporation. Then, equal volume of ethyl acetate was added to extract the products. Finally, ethyl acetate was removed under reduced pressure and extracts were dissolved in 2 mL methanol. The HPLC analysis was carried out with a linear gradient program over 30 min at a flow rate of 1 mL·min^−1^: (solvent A: H_2_O; solvent B: CH_3_CN), 0–5 min: 85% B; 5–10 min: 85%–95% B; 10–25 min: 95% B; 25–30 min: 95%–85% B. The LC-MS analysis was performed with a linear gradient program over 35 min at a flow rate of 0.6 mL·min^−1^: (solvent A: H_2_O; solvent B: CH_3_CN), 0–5 min: 72% B; 5–30 min: 72%–100% B; 30–33 min: 100% B; 33–35 min: 100%–72% B.

### 4.10. Purification of Compounds **3**–**5** and **10**

A total of 1 kg of rice medium [27] and 2 L PDA medium were used for the cultivation of Ao-*oblA_Au_*-*oblB_Au_*-*oblD_Au_* strains and mutant Δ*oblC_Au_*, respectively. The crude extract of strains was obtained using the above method and was separated on a silica gel column chromatography using stepwise gradient elution of petroleum ether–ethyl acetate (90/10-75/25) to provide the mixture of **3**–**5**. The mixture was further isolated by semi-preparative HPLC (Agilent 1260 Infinity, Agilent Technologies, Waldbronn, Germany) equipped with Innoval ODS-2 C18 column (10 × 250 mm, 5 μm, Agela Technologies, Delaware, USA). The isocratic elution program of CH_3_CN/H_2_O (85:15) was performed over 25 min at a flow rate of 3 mL·min^−1^.

Strain Ao-*oblA*-*oblC* was cultured in DPY plates and the crude extract from 2 L DPY medium was separated on a silica gel column chromatography using stepwise gradient elution of petroleum ether–ethyl acetate (100/0-90/10) to obtain compound **10**. Compound **10** was further purified by HPLC following the method of detecting ophiobolin F (**1**) at a flow rate of 3 mL·min^−1^.

### 4.11. Feeding Experiments in ΔoblA_Au_ Mutant

Mutant Δ*oblA_Au_* was inoculated at PDA medium in a 3.5 × 3.5 mm Petri dish. After cultivation for 24 h at 28 °C, more than 0.1 mg of compounds **1** or **10** was coated on the surface of PDA medium. The cultures were harvested after further cultivation for 4 d at 28 °C, extracted with acetone, concentrated and analyzed by HPLC. HPLC analysis was carried out with a linear gradient program of 45 min at a flow rate of 1 mL·min^−1^: (solvent A: H_2_O; solvent B: CH_3_CN), 0–15 min: 50%–100% B; 15–35 min: 100% B; 35–40 min: 100%–50% B; 40–45 min: 50% B.

### 4.12. Florescence Observation

The *egfp* gene was amplified from the plasmid peGFP-C1 with primers eGFP-F/A. Genes *oblA*-*D* were amplified from genomic DNA of *A. ustus* 094102 with primers *eGFP*-*oblA*-F/R, *eGFP*-*oblB*-F/R, *eGFP*-*oblC*-F/R and *eGFP*-*oblD*-F/R, respectively. Then, *egfp* was fused to the 5′ of *oblA*-*D*, respectively. Four fragments, *egfp*-*oblA*, *egfp*-*oblB*, *egfp*-*oblC* and *egfp*-*oblD,* were inserted into the site of *EcoR*I in pTAex3-rev-1022 to construct the plasmid pTAex3-*egfp-oblA*, pTAex3-*egfp-oblB*, pTAex3-*egfp-oblC* and pTAex3-*egfp-oblD*, respectively. Four plasmids of *A. oryzae* NSAR1 were individually transformed to obtain the transformants Ao-*egfp-oblA*, Ao-*egfp-oblB*, Ao-*egfp-oblC* and Ao-*egfp-oblD*. The transformant was cultured in liquid Czapek–Dox (CD) medium (0.3% NaNO_3_, 0.2% KCl, 0.05% MgSO_4_∙7H_2_O, 0.1% KH_2_PO_4_, 0.002% FeSO_4_∙7H_2_O, 1% polypeptone, supplement 3% of maltose, pH5.5) at 28 °C and shaken at 200 rpm for 3–5 d. For observation of florescence, fresh mycelium was washed three times with 1 mL sterilized water and then incubated with 100 μL DAPI Staining Solution (Beyotime Biotech, Shanghai, China) at room temperature for 10 min and washed with 1 mL sterilized water once. For Mito-Tracker Red CMXRos staining (Beyotime Biotech, Shanghai, China), 100 μL regent was added into the mycelium, washed by sterilized water and treated at 37 °C for 30 min. After being washed by 1 mL sterilized water once, 100 μL DAPI Staining Solution was added for nuclear staining. The staining of the mycelium was observed by using a fluorescence microscope (Axio Scope A1, ZEISS, Baden-Wurttemberg, Germany). The excitation/emission wavelengths used were from 488 nm to 550 nm for GFP, 364 nm to 454 nm for DAPI and 579 nm to 599 nm for red fluorescence signals.

### 4.13. Inhibition Tests on A. oryzae

To test the inhibition effect on *A. oryzae*, a series of concentrations (0, 0.1, 0.5, 1, 2, 5, 8, 10, 20 and 50 μM) of compounds **2** and **3** were prepared. The different concentrations of compounds were coated on CD medium in 3.5 × 3.5 mm Petri dish. Spores (~5 × 10^5^) were inoculated into the center of medium and cultured for 48 h at 28 °C. The diameters of *A. oryzae* colonies under the function of different concentrations of compounds **2** and **3** were measured. Three biological repeats were measured. The inhibition rate was calculated with the formula of (D_0_-D)/D_0_, D_0_ representing the diameter of colony in 0 μM of compounds; D represented the diameter of colony in 0.1-50 μM of compounds, respectively.

### 4.14. Cytotoxicity Assay

The MTT method was used to test cell proliferation. All cell lines were preserved in our lab [23,38]. MCF-7 and MDA-MB-231 were cultured in Dulbecco’s Modified Eagle Medium (DMEM, HyClone, Logan, Utah, USA) and MCF-7/ADR cells in Roswell Park Memorial Institute 1640 medium (RPMI 1640, HyClone, Logan, Utah, USA) in a 5% CO_2_ incubator at 37 °C. Cells were incubated in a 96-well plate with 8,000 cells per well for 24 h. Compounds **3**–**5** and **10** were diluted to a series of concentrations with medium and added to 96-well plates. Medium containing DMSO was set as control. After continuing to cultivate for 48 h, 5 mg·mL^−1^ MTT was added to each well; then, the culture medium was removed carefully and 100 μL DMSO was added for 4-h staining. The absorbance was measured at 490 nm. Additionally, the value of IC_50_ at the concentration of a 50% reduction in growth was calculated by SPSS analysis. Three biological repeats were measured.

### 4.15. Homology Modeling and Molecular Docking

The homology model of OblC_Au_ was built using the SWISS-MODEL web-based modeling server (https://www.swissmodel.expasy.org/, accessed on 1 November 2021) [41], which showed 16.35% sequence identity with spermidine dehydrogenase SpdH. Docking between the model and substrates was carried out using the AutoDock software [42]. The docking results were visualized by UCSF Chimera package [43].

## 5. Conclusions

In this research, we completely investigated the biosynthesis pathway of **2** in *A. ustus* 094102, and confirmed that the flavin-dependent oxidase OblC_Au_ was unclustered and displayed the feature of substrate promiscuity. For the first time, we performed the subcellular localization of proteins involved in ophiobolins biosynthesis, and demonstrated that the biosynthesis of ophiobolins is compartmentalized. Based on this information, the transporter OblD could transfer toxic intermediates or final products outside of the cell membrane for the self-protection of hosts. At the same time, we also realized the de novo synthesis of **2** in a heterologous host to build blocks for constructing high-yield ophiobolin engineering strains by using a combinatorial biosynthetic approach.

## Figures and Tables

**Table 1:** The IC_50_ values (μM) of compounds **3**–**5** and **10** on MCF-7, MDA-MB-231 and MCF-7/ADR cell lines.

	MCF-7	MDA-MB-231	MCF-7/ADR
**3**	4.88 ± 0.45	1.92 ± 0.67	0.77 ± 0.08
**4**	3.73 ± 0.16	1.52 ± 0.21	2.71 ± 0.31
**5**	3.64 ± 0.34	2.26 ± 0.15	1.82 ± 0.03
**10**	>10	7.32 ± 0.82	>20

## Data Availability

Not applicable.

## Supplementary Materials

The following supporting information can be downloaded at: https://www.mdpi.com/article/10.3390/ijms23031903/s1.
