# Supplementary material for: The Biosynthesis and Transport of Ophiobolins in Aspergillus ustus 094102
Source: Int J Mol Sci. 2022 Feb 8;23(3):1903. doi: 10.3390/ijms23031903 (PMC8836403; doi:10.3390/ijms23031903)
Supplement: Supplementary file 1 [file ijms-23-01903-s001.zip › ijms-1584171-supplementary.pdf]

Supporting Information

Table of Contents

Supplementary tables **3**

Table S1 Proposed function of genes in *obl*$_{Au}$ gene cluster 3

Table S2 Primers used in this study 3

Table S3 Putative FAD dependent oxidoreductase in *A. ustus* 094102 5

Table S4 ^{1}H NMR (400 MHz) data of compound **1**-**7** and **10** in $CDCl_3$ [1-3] 6

Table S5 ^{13}C NMR (100 MHz) data of compound **1**-**7** and **10** in $CDCl_3$ [1-3] 8

Table S6 Plasmids used in this study 8

Supplementary figures **10**

Figure S1 *obl* gene cluster from *A. ustus, A. stellatus, A. clavatus* and *Bipolaris maydis* [4, 5] 10

Figure S2 Gene expression level analysis of *oblA*$_{Au}$-*D*$_{Au}$ and *oblR*$_{Au}$ by RNA-Seq (a) and RT-qPCR (b) 10

Figure S3 Screening of mutants 11

Figure S4 PCR verification of heterologous expression strains 12

Figure S5 HPLC analysis of crude extracts from *A. oryzae* expressing *oblA*$_{Au}$-*D*$_{Au}$ 13

Figure S6 HPLC analysis of cultural extracts of mutants Δ*oblA*$_{Au}$ fed with compound **1** and **10** 13

Figure S7 The inhibition test of 0-50 μM of compound **2** and **3** on *A. oryzae* 14

Figure S8 Docking of the modelled OblC with different substrates 14

NMR and HR-ES spectrums **15**

Figure S9 ^{1}H NMR spectrum of compound **1** (400 MHz, $CDCl_3$) 15

Figure S10 ^{13}C NMR spectrum of compound **1** (100 MHz, $CDCl_3$) 15

Figure S11 ^{1}H NMR spectrum of compound **2** (400 MHz, $CDCl_3$) 16

Figure S12 ^{13}C NMR spectrum of compound **2** (100 MHz, $CDCl_3$) 16

Figure S13 ESI-HRMS spetrum of compound **2** 17

Figure S14 ^{1}H NMR spectrum of compound **3** (400 MHz, $CDCl_3$) 17

Figure S15 ^{13}C NMR spectrum of compound **3** (100 MHz, $CDCl_3$) 18

Figure S16 ESI-HRMS spetrum of compound **3** 18

Figure S17 ^{1}H NMR spectrum of compound **4** (400 MHz, $CDCl_3$) 19

Figure S18 ^{13}C NMR spectrum of compound **4** (100 MHz, $CDCl_3$) 19

Figure S19 ESI-HRMS spetrum of compound **4** 20

Figure S20 ^{1}H NMR spectrum of compound **5** (400 MHz, $CDCl_3$) 20

Figure S21 ^{13}C NMR spectrum of compound **5** (100 MHz, $CDCl_3$) 21

Figure S22 ESI-HRMS spetrum of compound **5** 21

Figure S23 ^{1}H NMR spectrum of compound **6** (400 MHz, $CDCl_3$) 22

Figure S24 ^{13}C NMR spectrum of compound **6** (100 MHz, $CDCl_3$) 22

Figure S25 ESI-HRMS spetrum of compound **6** 23

Figure S26 ^{1}H NMR spectrum of compound **7** (400 MHz, $CDCl_3$) 23

Figure S27 ^{13}C NMR spectrum of compound **7** (100 MHz, $CDCl_3$) 24

Figure S28 ESI-HRMS spetrum of compound **7** 24

Figure S29 ^{1}H NMR spectrum of compound **10** (400 MHz, $CDCl_3$) 25

Figure S30 ^{13}C NMR spectrum of compound **10** (100 MHz, $CDCl_3$) 25
Figure S31 ESI-HRMS spetrum of compound **10**..26
Figure S32 DEPT135 of compound **10** (100 MHz, $CDCl_3$)..26
Figure S33 ^{1}H-^{1}H COSY spectrum of compound **10** (400 MHz, $CDCl_3$) 27
Figure S34 HMBC spectrum of compound **10** (400 MHz, $CDCl_3$) 27
Figure S35 HSQC spectrum of compound **10** (400 MHz, $CDCl_3$) 28
Supplementary references 29

Supplementary tables

Table S1 Proposed function of genes in *obl*$_{Au}$ gene cluster

Gene	Size (aa)	Proposed function	Species	Identity (%)	Sequence ID
oblA$_{Au}$	725	Ophiobolin F synthase OblA	*A. stellatus*	82.28	A0A1V1FVQ6.1
			A. clavatus NRRL 1	65.34	XP_001276070.1
			B. maydis C5	61.37	M2V8C1.1
oblB$_{Au}$	544	Cytochrome P450 monooxygenase OblB	*A. stellatus*	79.38	A0A1V1FNM9.1
			A. clavatus NRRL 1	66.22	XP_001276069.1
			B. maydis C5	66.79	M2V933.1
oblD$_{Au}$	1464	ABC transporter OblD	*A. stellatus*	85.64	A0A1V1GB10.1
			A. clavatus NRRL 1	78.04	XP_001276075.1
			B. maydis C5	74.90	M2UCE5.1
oblR$_{Au}$	426	Putative Zn (II)$_2$Cys$_6$ transcription factor	*A. nidulans* FGSC A4	21.96	CBF83040.1
			A. stellatus	50.54	BAX09284.1

Table S2 Primers used in this study

Purpose	Primers	Sequence (5' to 3')
For qPCR	q-*oblA*-s	ATATTGTGGCTCTCAGCCTCG
	q-*oblA*-a	CTGAGCCATGTGTTGAGCG
	q-*oblB*-s	CCGTGACAGGCTACTACGAG
	q-*oblB*-a	GTCGCGGATGGAGAGTTCAA
	q-*oblC*-s	TGTCAGACCACTCGACCTCA
	q-*oblC*-a	CCAAGGCACCAGCGTAGTAA
	q-*oblD*-s	CAATTATCAAGGCGCGACTGC
	q-*oblD*-a	GTATCTCCAACGGTGAGTCTGG
	q-*oblR*-s	GGAACCTCCAGACTACAGCG
	q-*oblR*-a	GGCGGATCAAGCTCTGCTAA
sh ble	*sh ble*-up	ATCGATGGGGACTAGTGATTAAGTGAGACCTTCGTTTGTGC
	sh ble-down	CTCACATGTTGGTCTCCAGCTTG
hph	*hph*-s	AAATTGACGCTTAGACAACTTAA
	hph-a	GCAGCTTGCCAACATGGTG
oblA gene deletion	Δ*oblA*-L-s	GAGACCAACATGTGAGAATCTCTAGACGAGTTGATGGTGGAGACG
	Δ*oblA*-L-a	AGTTGTCTAAGCGTCAATTTGTCGAGTACTTATACTCCAT
	Δ*oblA*-R-s	CCACCATGTTGGCAAGCTGCTGGAGCTGCTGAAGGTTTGA
	Δ*oblA*-R-a	GTTGTAAAACGACGGCCAGTGAATTCTGCCGAGTTATTAGTATGGA
	Δ*oblA*-yz-s	GTCTCAGCTCGACACCACA
	Δ*oblA*-yz-a	GCACCCAATCCAATGTTT
oblB gene deletion	Δ*oblB*-L-s	GTCGACGATTATCGATGGGGACTAGTCACATCTCCACCTCCCTC
	Δ*oblB*-L-a	TACGGGACGGACGAGTCGGA
	oblB-*hph*-s	TCCGACTCGTCCGTCCCGTAAAATTGACGCTTAGACAACTTAA

	Δ*oblB*-R-s	CCACCATGTTGGCAAGCTGCCGCCTGGGATGGTAGAAG
	Δ*oblB*-R-a	GTTGTAAAACGACGGCCAGTGAATTCCTTGGCATCAGCGTGGTA
	Δ*oblB*-yz-s	TCCACCCACGAACTCATC
	Δ*oblB*-yz-a	CCGACGCATCCATTGTAG
oblC gene deletion	Δ*oblC*-L-s	GCTATGACCATGATTACGCCAAGCTTATTGAGAAGGAGGAGTTGG
	Δ*oblC*-L-a	AGTTGTCTAAGCGTCAATTTGGCTGGACGAAGTAAGTAGA
	Δ*oblC*-R-s	CCACCATGTTGGCAAGCTGCAATCTTTTGTGGTTTCTTTTGGC
	Δ*oblC*-R-a	GTTGTAAAACGACGGCCAGTGAATTCCACAGACCTTGTTGCTCC
	Δ*oblC*-yz-s	CCCATTCTTAGTCAGTTCTTG
	Δ*oblC*-yz-a	CCTCTTCGGTCTTGTGGTA
oblD gene deletion	Δ*oblD*-L-s	GAATCTCTAGAGGATCCCCGGGTACCGTGGATAAGCAGAGGCAATA
	Δ*oblD*-L-a	AGTTGTCTAAGCGTCAATTTGACGGATGTCAAGAAGAGC
	Δ*oblD*-R-s	CCACCATGTTGGCAAGCTGCTACCGTCGTCGAAATCGT
	Δ*oblD*-R-a	GTTGTAAAACGACGGCCAGTGAATTCGAGGGAGGTGGAGATGTGAG
	Δ*oblD*-yz-s	GAGGCTATGCGTATGTTGC
	Δ*oblD*-yz-a	CTTCTGCCGTTTGGATGG
oblR gene deletion	Δ*oblR*-L-s	GTCGACGATTATCGATGGGGACTAGTTGAGGAAGGTGGAGCAGC
	Δ*oblR*-L-a	AGTTGTCTAAGCGTCAATTTGGTGAGACGAGCCGTGTTT
	Δ*oblR*-R-s	CCACCATGTTGGCAAGCTGCTTTGAATTGCATCGAATAGGGG
	Δ*oblR*-R-a	GTTGTAAAACGACGGCCAGTGAATTCGCCCGTCATGAAGGACTAT
	Δ*oblR*-yz-s	GCTGAGGAACGACCAATC
	Δ*oblR*-yz-a	GACGTTTGGTCGCTGATC
For heterologous expression in *A. oryzae*	AO-*oblA*-F	TAAACCCCACAGCAAGCTCCGAATTCATGGAGTATAAGTACTCGAC
	AO-*oblA*-R	TTCACGAGCTACTACAGATCCCCGGGTCAAACCTTCAGCAGCTCCA
	AO-*oblB*-F	TAAACCCCACAGCAAGCTCCGAATTCATGGAGGCCTACCTGCCCCAA
	AO-*oblB*-T$_{oblB}$-R	AAATGATCAAAACACCATGAAGATGTTCAGCGCCACGT
	Pamy-F	TGACGTGGCGCTGAACATCTTCATGGTGTTTTGATCAT
	Pamy-R	GTCGAGTACTTATACTCCATCTGTGGGGTTTATTGTTCAG
	Adea-Pamy-F	GTTGCGCAGATATCCATATGACTAGTTCATGGTGTTTTGATCAT
	Pamy-R’	TTCACGAGCTACTACAGATCTCACTCCTTCTTGCCATCGC
	Tamy-F	GCGATGGCAAGAAGGAGTGAGATCTGTAGTAGCTCGTGAA
	Adea-Tamy-R	TTGCATGCCTGCAGGTCGACTCTAGACCATCGATGGATCTCCTTTTGCTTTCTGCC
	AO-*oblD*-F	TAAACCCCACAGCAAGCTCCGAATTCATGGCCACATCAAACGATTTC
	AO-*oblD*-R	TTCACGAGCTACTACAGATCTCACTCCTTCTTGCCATCGC
	AO-*oblC*-F	GTTGCGCAGATATCCATATGACTAGTTCATGGTGTTTTGATCAT
	AO-*oblC*--T$_{oblC}$ R	TTGCATGCCTGCAGGTCGACTCTAGAAGGATGCACAAGGCAACGGT

	Pamy-*oblC*-F	AAAAGGAGATCCATCGATGGTCTAGATCATGGTGTTTGATCAT
	Pamy-*oblC*-R	AAAAGAAACCACAAAAGATTTCATAGGCCCTCAACAAG
eGFP	eGFP-F	TAAACCCCACAGCAAGCTCCGAATTCATGGTGAGCAAGGGCGAGGA
	eGFP-A	CTTGTACAGCTCGTCCATGC
oblA	eGFP-*oblA*-F	GCATGGACGAGCTGTACAAGATGGAGTATAAGTACTCGAC
	eGFP-*oblA*-R	GATCCCCGGGTACCGAGCTCGAATTCTCAAACCTTCAGCAGCTCCA
oblB	eGFP-*oblB*-F	GCATGGACGAGCTGTACAAGATGGAGGCCTACCTGCCCCAA
	eGFP-*oblB*-R	GATCCCCGGGTACCGAGCTCGAATTCTCAATGGTACACAACGCGAA
oblC	eGFP-*oblC*-F	GCATGGACGAGCTGTACAAGATGCCTCTCCCTAAATCAT
	eGFP-*oblC*-R	GATCCCCGGGTACCGAGCTCGAATTCTCATAGGCCCTCAACAAGCG
oblD	eGFP-*oblD*-F	GCATGGACGAGCTGTACAAGATGGCCACATCAAACGATTT
	eGFP-*oblD*-R	GATCCCCGGGTACCGAGCTCGAATTCTCACTCCTTCTTGCCATCGC
Partial of *arg*	Arg-S	ATCTCATACCCGTCAACTCA
gene	Arg-A	ACTCGCCTCATCCGTCAT
Partial of *ade*	Ade-S	AATGACTTGGGTAAGACGG
gene	Ade-A	CTGAATGGGCTAAGGAGA

Table S3 Putative FAD dependent oxidoreductase in *A. ustus* 094102

Genes	Identity	FPKM in ophs (+)	FPKM in ophs (-)
au-orf1	58%	378.81	2.35
au-orf2	37%	3.26	3.56
au-orf3	33%	4.11	34.4

Note: oph(+), ophiobolin-producing strain; oph(-), ophiobolin-nonproducing strain; FPKM, Fragments Per Kilobase of exon model per Million mapped fragments.

Table S4 ^{1}H NMR (400 MHz) data of compound **1**-**7** and **10** in $CDCl_3$ [1-3]

Position	δH (*J* in Hz)							
	**1**	**2**	**3**	**4**	**5**	**6**	**7**	**10**
1	1.86 m, 1.94 m	1.20 (d, 14.6), 1.79 (d, 2.9)	1.24 m, 1.81 (dd, 14.6, 3.2)	1.56 m, 1.76 m	1.16 m, 2.04 m	1.59 m, 1.80 m	2.04 (d, 3.6), 1.18 (t, 13.1)	1.19 m, 1.87 m
2	1.60 m	2.37 (dt, 19.0, 4.4)	2.39 m	2.14 m	2.67 m	2.12 m	2.68 m	1.67 m
4	1.68 m	2.51 (d, 19.2), 2.81 (d, 19.2)	2.50 (d, 19.2), 2.80 (d, 19.3)	2.43 (dd, 16.8, 1.5), 3.08 (d, 16.7)	6.03 (t, 1.6)	2.42 (dd, 16.7, 1.6)	6.06 s	1.74 m, 1.91 m
5	1.39 m, 1.52 m							1.36 m, 1.53 m
6	3.17 (t, 8.2)	3.28 (d, 10.5)	3.27 (d, 10.5)	3.35 (d, 10.8)	3.44 (d, 4.2)	3.30 (d, 10.6)	3.41 (d, 3.8)	3.14 (t, 8.4)
8	5.53 (tt, 8.2, 1.5)	7.14 (t, 8.6)	7.22 (t, 8.5)	6.89 (dd, 6.7, 2.2)	6.84 (dd, 6.3, 2.4)	6.87 (dd, 6.8, 2.3)	6.84 (d, 4.3)	6.00 (dt, 11.5, 1.3)
9	1.99 m	2.97 (dd, 12.6, 8.3), 2.13 (dd, 12.5, 7.6)	2.29 m, 2.44 m	2.65 m, 2.21 m	2.70 m, 2.26 m	2.17 (d, 14.6), 2.83 (dt, 20.5, 3.2)	2.94 (d, 20.4), 2.25 m	1.83 m, 2.02 (qd, 9.6, 6.9)
10	1.68 m	1.57 m	-	2.60 m	2.70 m	2.51 m	2.64 m	1.93 m
12	1.33 m, 1.49 m	1.42 m, 1.44 m	1.39 m, 1.46 m	1.41 m, 1.47 m	1.40 m, 1.49 m	1.47 m, 1.50 m	1.55 m, 1.46 (td, 12.3, 4.9)	1.44 (dd, 13.6, 3.2), 1.52 m
13	1.25 m, 1.49 m	1.25 m, 1.61 m	1.43 m, 1.54 m	1.16 (qd, 12.2, 5.8), 1.56 m	1.24 m, 1.58 m	1.20 m, 1.68 m	1.27 m, 1.69 m	1.33 m, 1.74 m
14	2.27 (dtd, 13.3, 8.8, 4.4)	2.08 (t, 9.5)	2.35 m	1.76 m	1.73 m	1.89 m	1.91 m	2.27 (dd, 13.9, 8.1)
15	1.68 m	2.71 (dt, 15.9, 8.0)	1.65 m	1.49 m	1.40 m	2.55 m	2.58 m	2.70 (ddt, 9.6, 6.6, 4.8)
16	1.21 m, 1.33 m	5.20 (t, 10.1)	1.16 m, 1.24 m	0.98 (tt, 9.2, 4.8), 1.38 m	0.98 m, 1.40 m	5.13 (t, 10.0)	5.13 (t, 10.0)	5.21 (t, 9.7)
17	1.91 m, 1.94 m	6.05 (t, 11.0)	1.95 m, 2.00 m	1.91 m, 2.05 m	1.92 m, 1.92 m	6.09 (t, 11.1)	6.12 (t, 11.1)	5.96 m
18	5.10 (dddd, 8.7,	5.98 (d, 11.7)	5.09 (tt, 7.0, 1.6)	5.10 m	5.10 m	6.00 (d, 11.6)	6.03 (d, 12.1)	5.47 (tt, 8.3, 1.5)

	7.2, 2.9, 1.5)							
20	1.27 s	1.37 s	1.36 s	1.44 s	2.06 s	1.44 s	2.09 s	1.26 s
21	1.80 s	9.22 s	9.23 s	9.19 s	9.30 s	9.16 s	9.29 s	1.71 s
22	0.87 s	0.98 s	0.90 s	0.83 s	0.85 s	0.84 s	0.88 s	0.92 s
23	0.76 (d, 6.8)	0.92 (d, 6.6)	0.78 (d, 6.8)	0.89 (d, 6.5)	0.89 (d, 6.5)	0.97 (d, 6.7)	0.99 (d, 6.7)	0.87 (d, 6.7)
24	1.60 s	1.75 s	1.60 s	1.60 s	1.60 s	1.76 s	1.79 s	1.77 s
25	1.68 s	1.82 s	1.69 s	1.69 s	1.69 s	1.82 s	1.85 s	1.80 s

Table S5 ^{13}C NMR (100 MHz) data of compound **1**-**7** and **10** in $CDCl_3$ [1-3]

Position	δC							
	**1**	**2**	**3**	**4**	**5**	**6**	**7**	**10**
1	26.24	35.09	36.21	41.39	45.90	41.40	45.95	26.60
2	54.29	50.28	51.05	49.62	49.08	49.68	49.23	53.32
3	81.28	76.89	76.97	76.76	177.68	76.87	177.62	80.97
4	42.05	54.91	54.99	55.01	130.33	55.05	130.39	41.65
5	23.78	217.64	217.73	217.34	207.56	217.40	207.67	25.73
6	42.51	48.54	48.66	48.96	50.09	48.92	50.07	41.93
7	136.60	141.33	141.65	141.83	140.46	141.41	140.08	138.25
8	129.67	164.06	164.36	159.93	157.16	160.96	158.08	134.78
9	23.70	25.44	24.99	31.06	31.11	30.95	31.00	24.31
10	56.00	53.56	53.64	43.14	43.19	43.85	43.93	55.77
11	44.09	43.85	44.09	44.44	45.15	44.81	45.50	43.59
12	43.69	42.59	42.69	45.59	44.68	45.36	44.42	43.30
13	36.90	26.55	23.03	27.31	27.21	27.82	27.87	35.77
14	45.87	47.13	45.46	51.66	51.60	52.17	52.18	47.38
15	33.41	35.85	32.96	32.03	31.95	32.69	32.69	35.53
16	37.78	137.11	37.09	37.00	37.30	135.70	135.81	121.55
17	26.71	122.44	26.20	25.87	25.83	124.09	124.13	120.54
18	125.46	119.95	124.65	124.57	124.51	120.08	120.08	129.02
19	131.58	136.06	131.58	131.69	131.72	136.57	136.65	136.02
20	29.49	25.67	25.68	25.92	17.36	25.99	17.34	29.05
21	21.97	196.28	196.49	194.45	193.17	194.43	193.13	21.58
22	19.25	18.73	19.18	23.56	23.19	23.43	23.04	20.54
23	17.39	20.42	16.68	18.75	18.72	21.43	21.35	18.22
24	18.14	18.15	17.80	17.84	17.83	18.34	18.32	18.59
25	26.31	26.55	25.87	25.83	25.88	26.69	26.65	26.63

Table S6 Plasmids used in this study

Plasmids	Features
pGAPZαA	*ble*^{r} selection marker
pCAMBIA1301	*hph* selection marker; CAMV35S promoter
pTAex3-rev-1022	*argB* auxotrophic gene; α-amylase promoter and terminator
pAdeA-R	*adeA* auxotrophic gene
peGFP-C1	*kan*^{r} and *neo*^{r} selection marker; *egfp* gene
pMD19T-*sh ble*	Introducing *Spe*Iand *Cla*Iinto multiple cloning sites of pMD19T
pMD19T-*oblA*L-*hph*-*oblA*R	*oblA* gene-targeting cassette inserted into *Xba*I and *EcoR*I
pMD19T-*oblB*L-*hph*-*oblB*R	*oblB* gene-targeting cassette inserted into *Spe*I and *EcoR*I
pMD19T-*oblC*L-*hph*-*oblC*R	*oblC* gene-targeting cassette inserted into *Hind* III and *EcoR*I
pMD19T-*oblD*L-*hph*-*oblD*R	*oblD* gene-targeting cassette inserted into *Kpn*I and *EcoR*I
pMD19T-*oblR*L-*hph*-*oblR*R	*oblR* gene-targeting cassette inserted into *Spe*I and *EcoR*I
pTAex3-*oblA*	*oblA* was introduced into between Pamy and Tamy

pAdeA-*oblB*	Introducing gene-expression cassette Pamy-*oblB*-Tamy
pAdeA-*oblC*	Introducing gene-expression cassette Pamy-*oblC*-Tamy
pAdeA-*oblD*	Introducing gene-expression cassette Pamy-*oblD*-Tamy
pTAex3-*oblA*-*oblB*	Introducing gene-expression cassette Pamy-*oblB*-T_{oblB}-Pamy-*oblA*-Tamy
pAdeA-*oblC*-*oblD*	Introducing gene-expression cassette Pamy-*oblC*-T_{oblC}-Pamy-*oblD*-Tamy
pTAex3-eGFP	*egfp* inserted into site of *EcoR* I
pTAex3-eGFP*oblA*	*egfp-oblA* inserted into site of *EcoR* I
pTAex3-eGFP*oblB*	*egfp-oblB* inserted into site of *EcoR* I
pTAex3-eGFP*oblC*	*egfp-oblC* inserted into site of *EcoR* I
pTAex3-eGFP*oblD*	*egfp-oblD* inserted into site of *EcoR* I

Supplementary figures

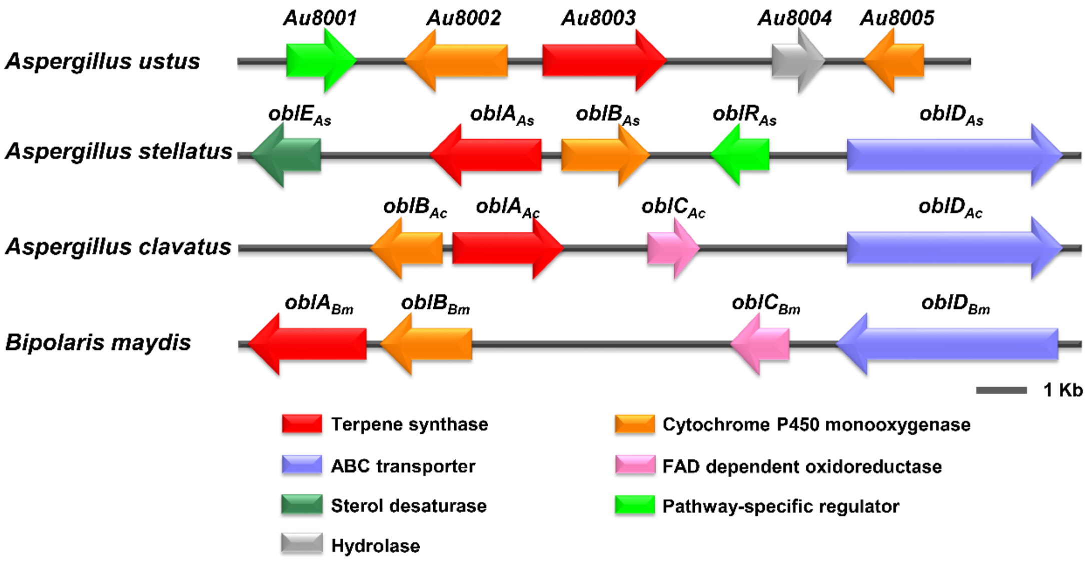

Figure S1 *obl* gene cluster from *A. ustus*, *A. stellatus*, *A. clavatus* and *Bipolaris maydis* [4, 5]

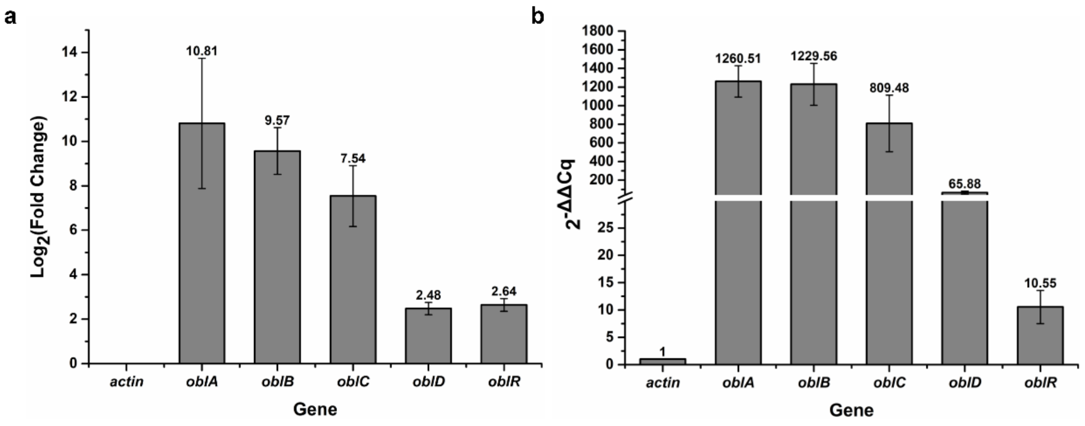

Figure S2 Gene expression level analysis of *oblA*$_{Au}$-*D*$_{Au}$ and *oblR*$_{Au}$ by RNA-Seq (a) and RT-qPCR (b). Log$_2$ (Fold Change) and $2^{-\Delta\Delta Cq}$ were calculated according to the expression ratio of each gene. The housekeeping gene *actin* was used for normalization. Data are means ± SE of three independent measurements.

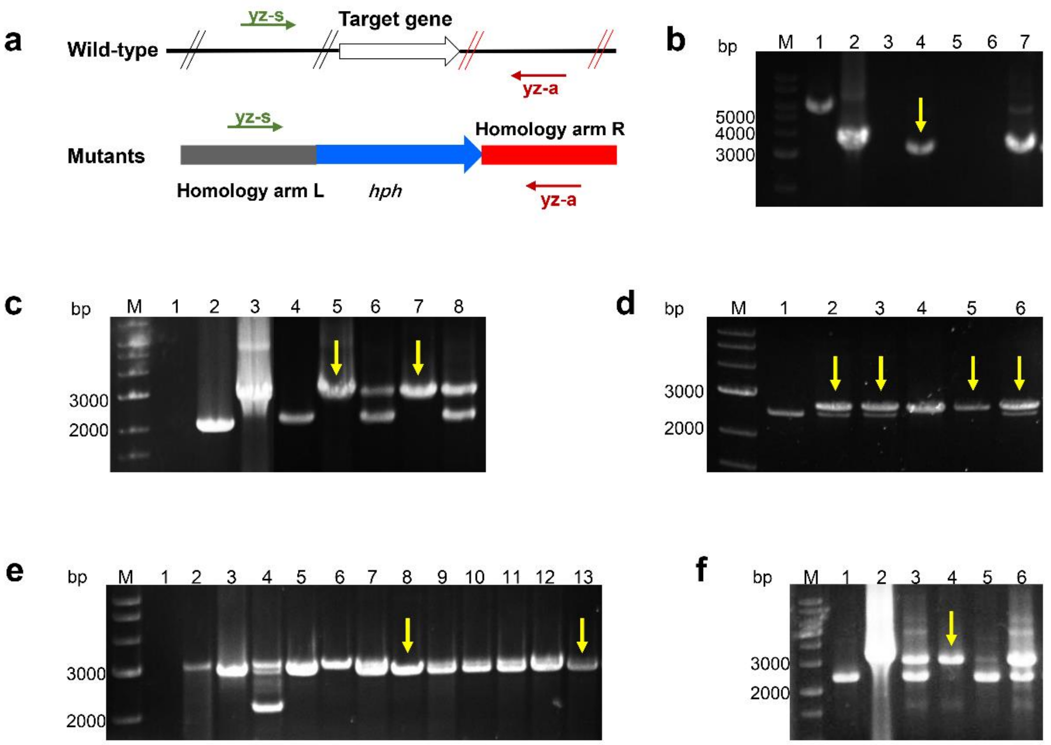

Figure S3 Screening of mutants. (a) The schematic diagram of screening of wild-type and mutants using primers yz-s/a. (b) PCR verification of Δ*oblD* transformants with primers *oblD*-yz-s/a. Lane 1: wild-type strain (5478 bp); lane 2: pMD19T-*oblD*L-*hph*-*oblD*R (3205 bp); lane 3: blank control; lane 4-7: four transformants. (c) PCR verification of Δ*oblR* transformants with primers *oblR*-yz-s/a. Lane 1: blank control; lane 2: wild-type strain (1999 bp); lane 2: pMD19T-*oblR*L-*hph*-*oblR*R (2805 bp); lane 4-8: five transformants. (d) PCR verification of Δ*oblB* transformants with primers *oblB*-yz-s/a. Lane 1: wild-type strain (2508 bp); lane 4: pMD19T-*oblB*L-*hph*-*oblB*R (2718 bp); lane 2-3 and 5-6: four transformants. (e) PCR verification of Δ*oblA* transformants with primers *oblA*-yz-s/a. Lane 1: blank control; lane 2: wild-type strain (3110 bp); lane 3: pMD19T-*oblA*L-*hph*-*oblA*R (2906 bp); lane 4-13: ten transformants. (f) PCR verification of Δ*oblC* transformants with primers *oblC*-yz-s/a. Lane 1: wild-type strain (2406 bp); lane 2: pMD19T-*oblC*L-*hph*-*oblC*R (3103 bp); lane 3-6: four transformants. M: 1 kb DNA ladder; the yellow arrows indicate positive mutants.

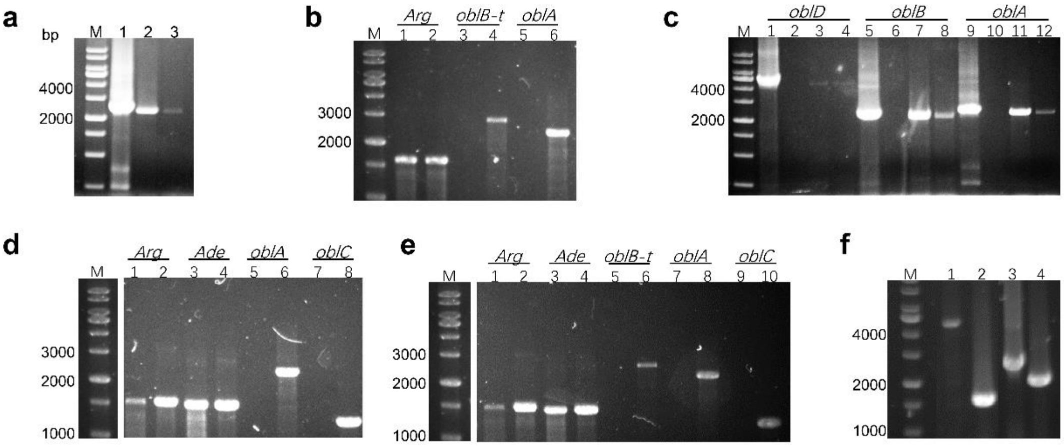

Figure S4 PCR verification of heterologous expression strains. (a) Ao-*oblA*. Lane 1, 2 and 3: oblA gene amplified from plasmid of pTAex3-*oblA*, gDNA of Ao-*oblA*-1 and gDNA of Ao-*oblA*-2, respectively; (b) Ao-*oblA*-*oblB*. Lane 1, 3 and 5: gDNA of Ao-pTAex3; Lane 2, 4 and 6: gDNA of Ao-*oblA*-*oblB*; (c) Ao-*oblA*-*oblB*-*oblD*. Lane 1: plasmid of pAdeA-oblD; Lane 5 and 9: plasmid of pTAex3-oblA-oblB; Lane 2, 6 and 10: gDNA of Ao-pTAex3-pAdeA; Lane 3, 7 and 11: gDNA of Ao-*oblA*-*oblB*-*oblD*-1; Lane 4, 8 and 12: gDNA of Ao-*oblA*-*oblB*-*oblD*-2. (d) Ao-*oblA*-*oblC*. Lane 1, 3, 5 and 7: gDNA of Ao-pTAex3-pAdeA; Lane 2, 4, 6 and 8: gDNA of Ao-*oblA*-*oblC*; (e) Ao-*oblA*-*oblB*-*oblC*. Lane 1, 3, 5, 7 and 9: gDNA of Ao-pTAex3-pAdeA; Lane 2, 4, 6, 8 and 10: gDNA of Ao-*oblA*-*oblB*-*oblC*; (f) Ao-*oblA*-*oblB*-*oblC*-*oblD*. Lane 1, 2, 3 and 4: *oblD*, *oblC*, *oblB-t* and *oblA* gene amplified from gDNA of Ao-*oblA*-*oblB*-*oblC*-*oblD*, respectively. M: 1 kb DNA ladder.

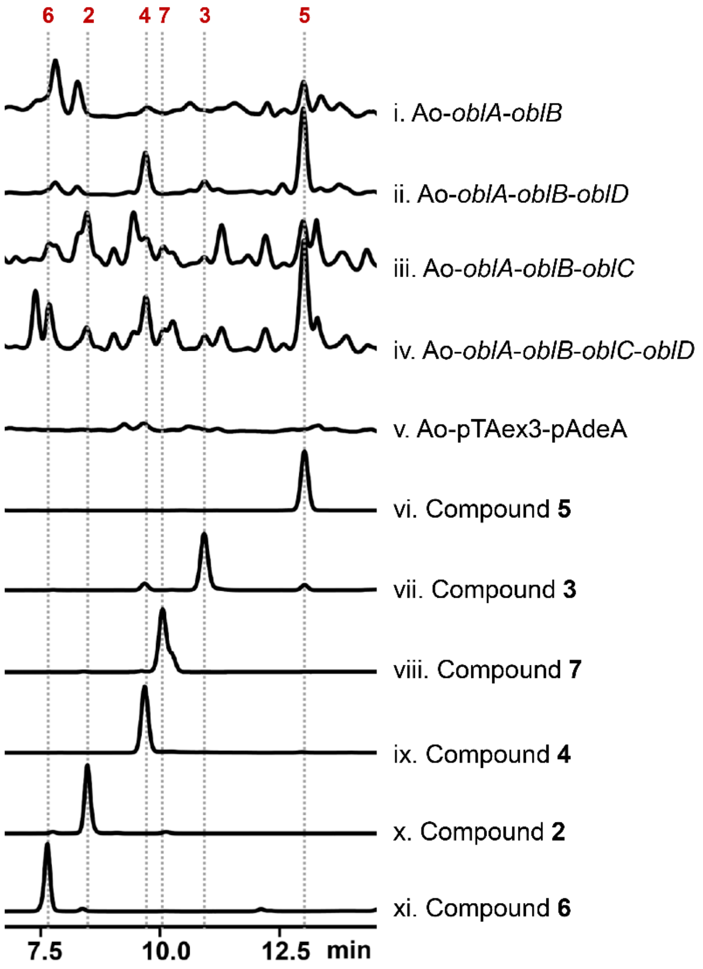

Figure S5 HPLC analysis of crude extracts from *A. oryzae* expressing *oblA*$_{Au}$-*D*$_{Au}$

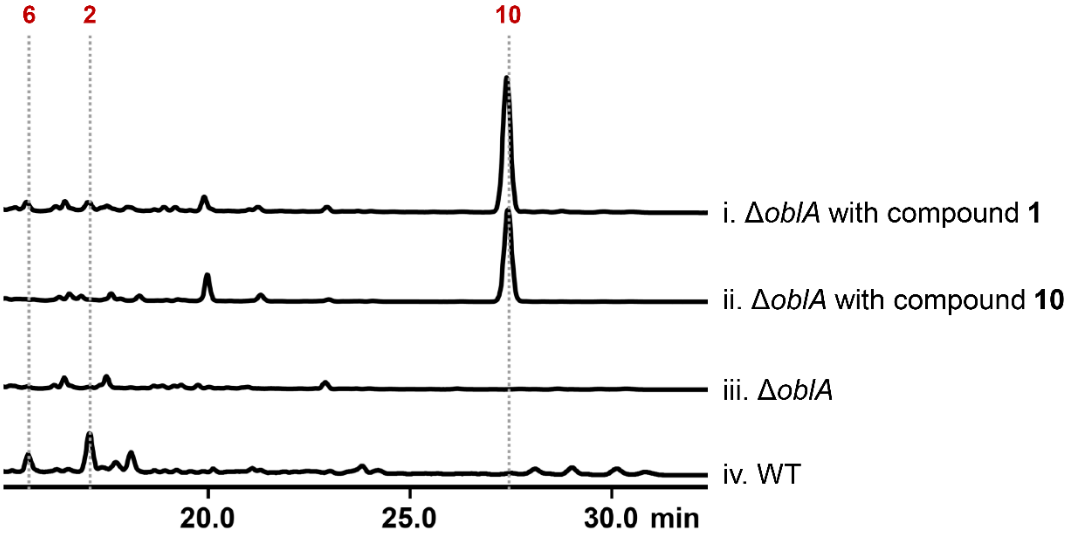

Figure S6 HPLC analysis of cultural extracts of mutants Δ*oblA*$_{Au}$ fed with compound **1** and **10**

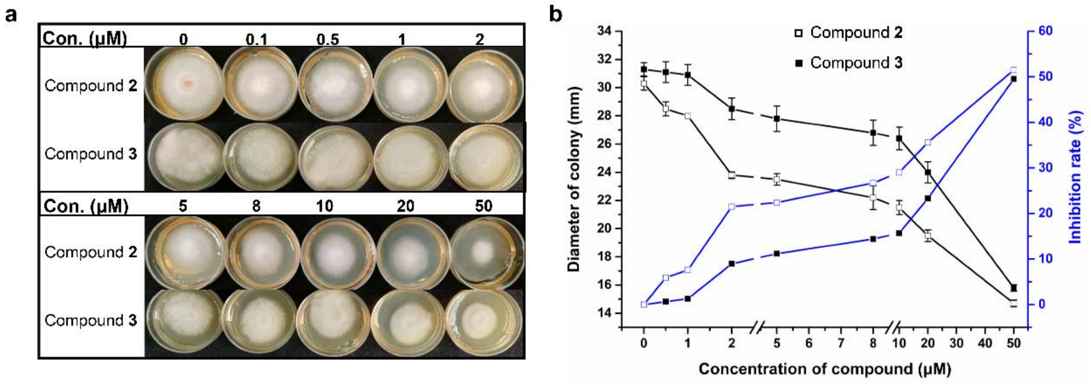

Figure S7 The inhibition test of 0-50 μM of compound **2** and **3** on *A. oryzae.* (a) The colony of *A. oryzae* growing in CD medium with 0-50 μM of compound **2** and **3**, respectively. (b) The diameter of colony of *A. oryzae* on CD medium with 0-50 μM of compound **2** and **3** and inhibition rate of 0-50 μM of compound **2** and **3** against *A. oryzae*, respectively.

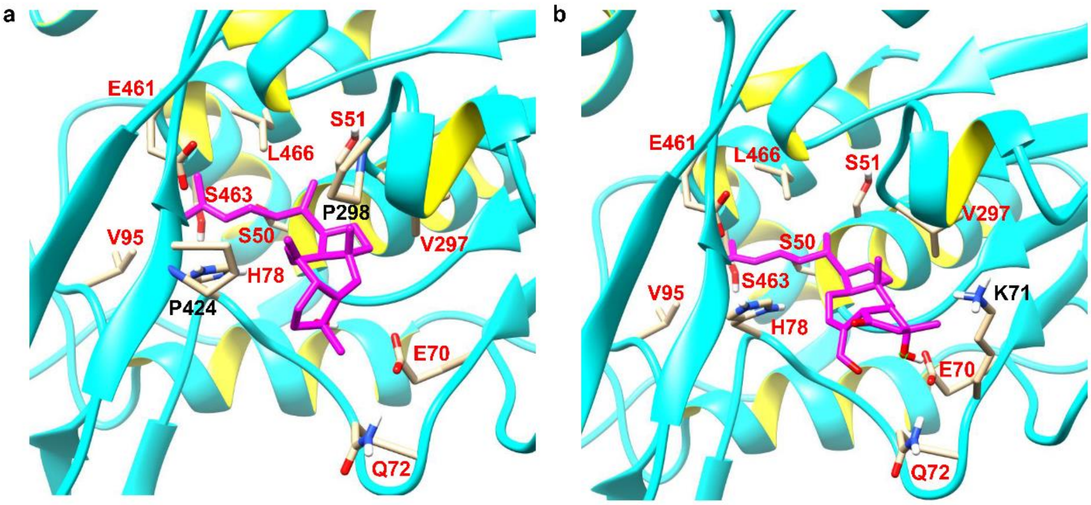

Figure S8 Docking of the modelled OblC with different substrates. (a) Docking of OblC with compound **1** (as sticks in lavender). The residues (S50, S51, E70, Q72, H78, V95, V297, V298, P424, E461, S463 and L466) are shown as sticks. (b) Docking of OblC with compound **3** (as sticks in lavender). The residues (S50, S51, E70, K71, Q72, H78, V95, V297, E461, S463 and L466) are shown as sticks. The residues in red are consensus residues in both models.

Supplementary NMR and HR-ES spectrums

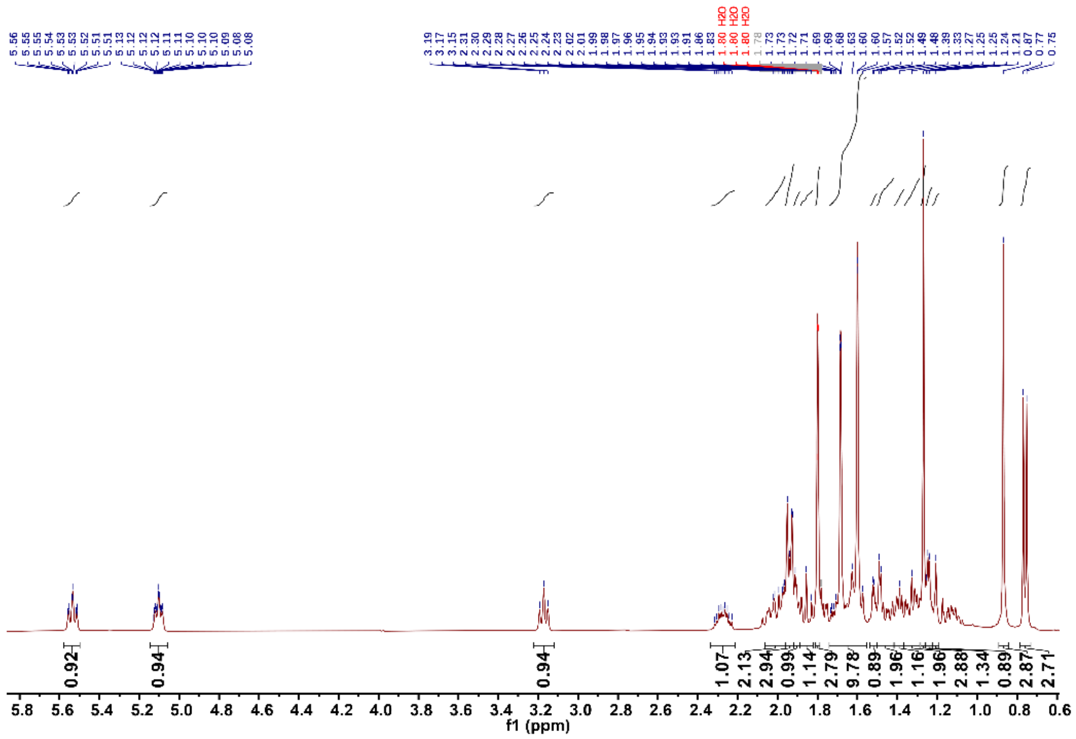

Figure S9 ^{1}H NMR spectrum of compound **1** (400 MHz, $CDCl_3$)

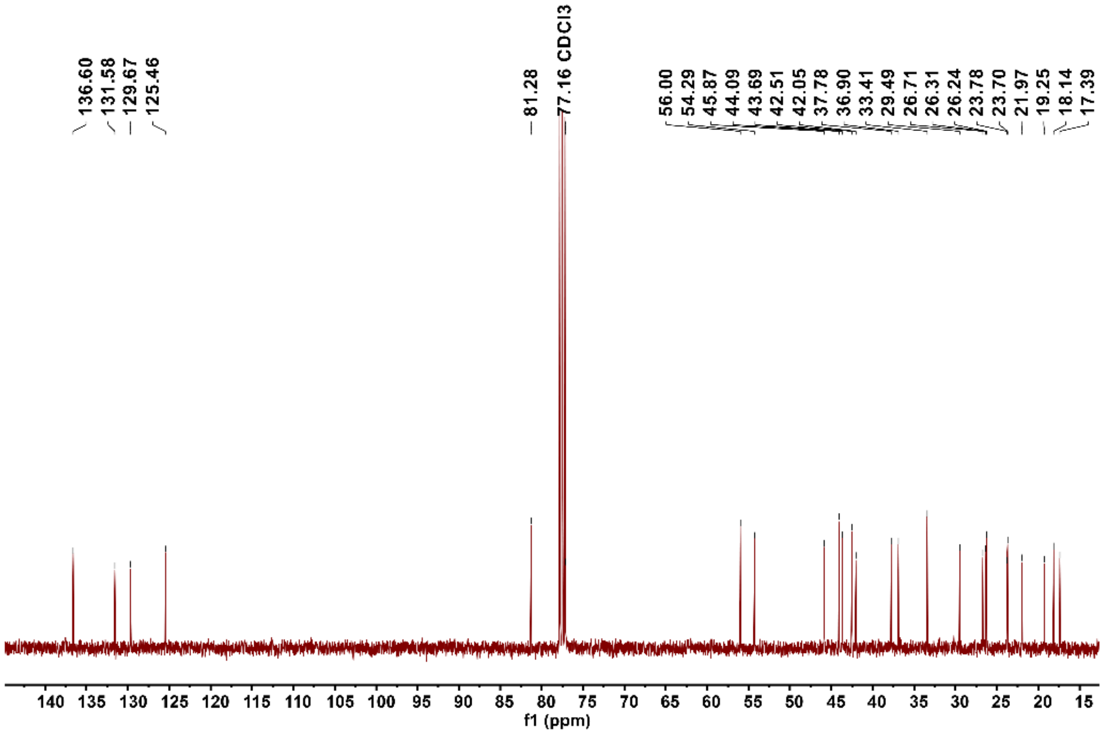

Figure S10 ^{13}C NMR spectrum of compound **1** (100 MHz, $CDCl_3$)

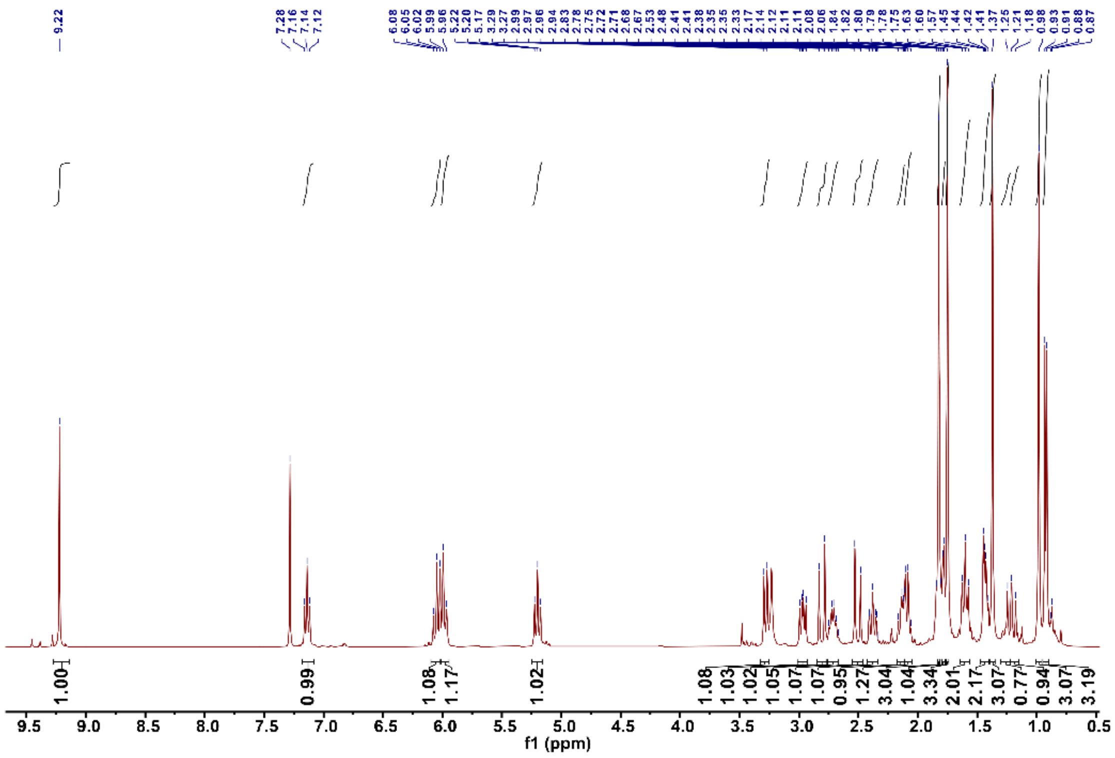

Figure S11 ^{1}H NMR spectrum of compound **2** (400 MHz, $CDCl_3$)

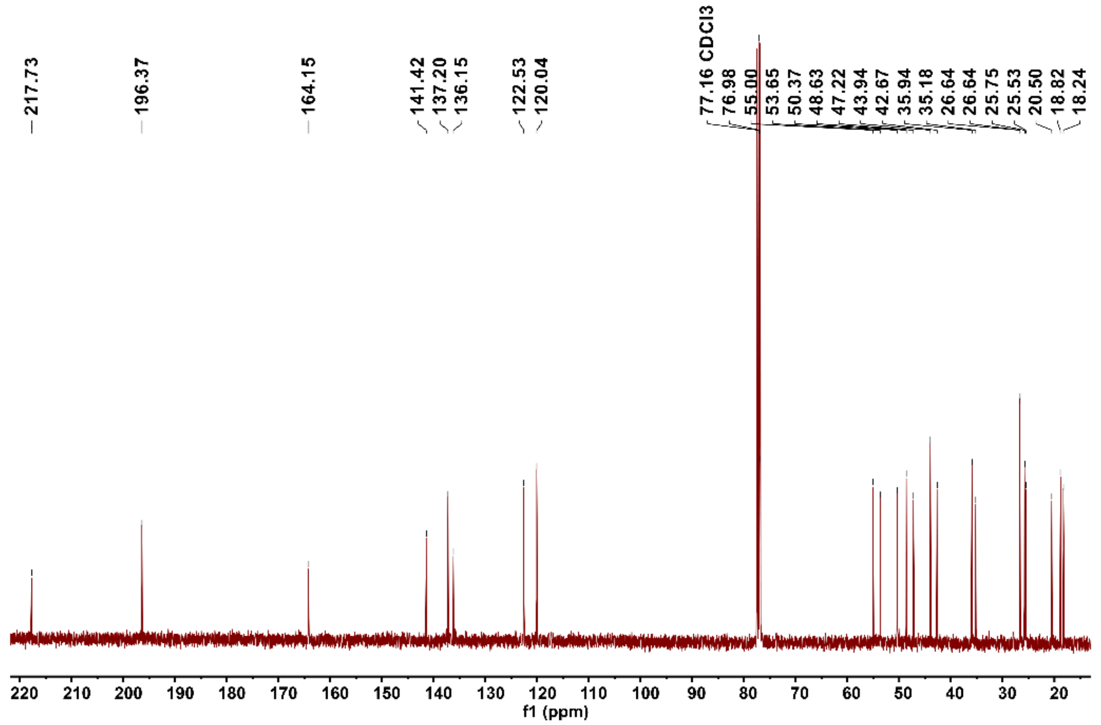

Figure S12 ^{13}C NMR spectrum of compound **2** (100 MHz, $CDCl_3$)

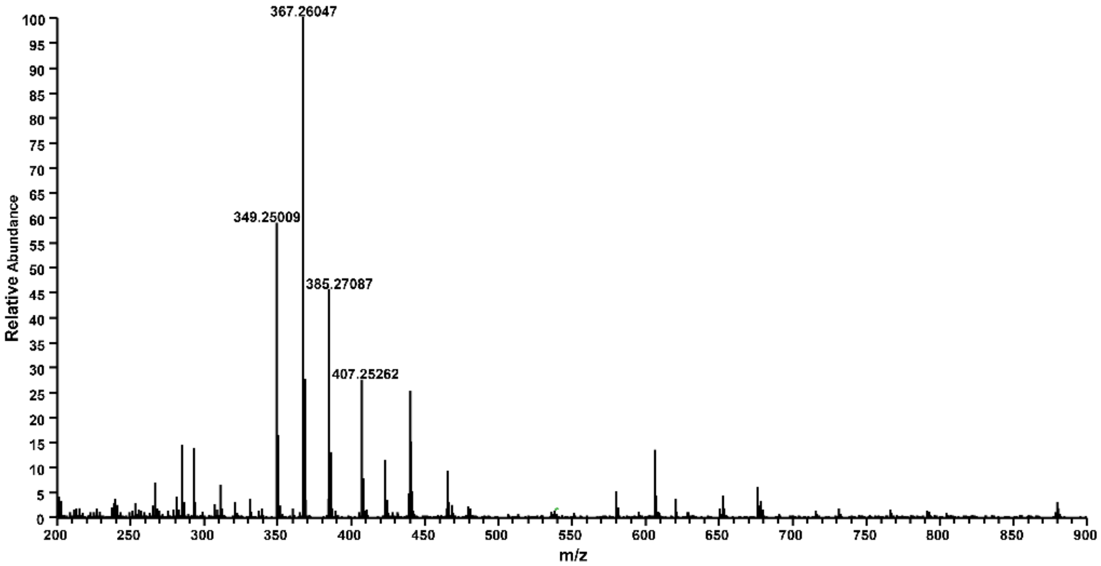

Figure S13 ESI-HRMS spetrum of compound **2**

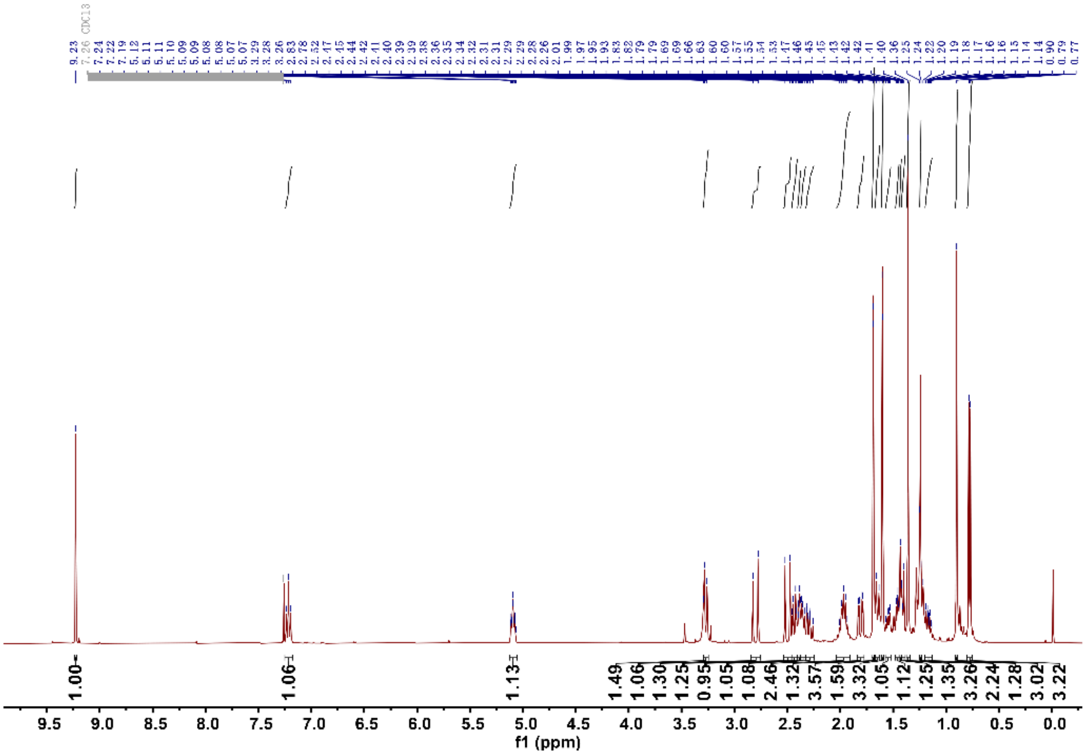

Figure S14 ^{1}H NMR spectrum of compound **3** (400 MHz, $CDCl_3$)

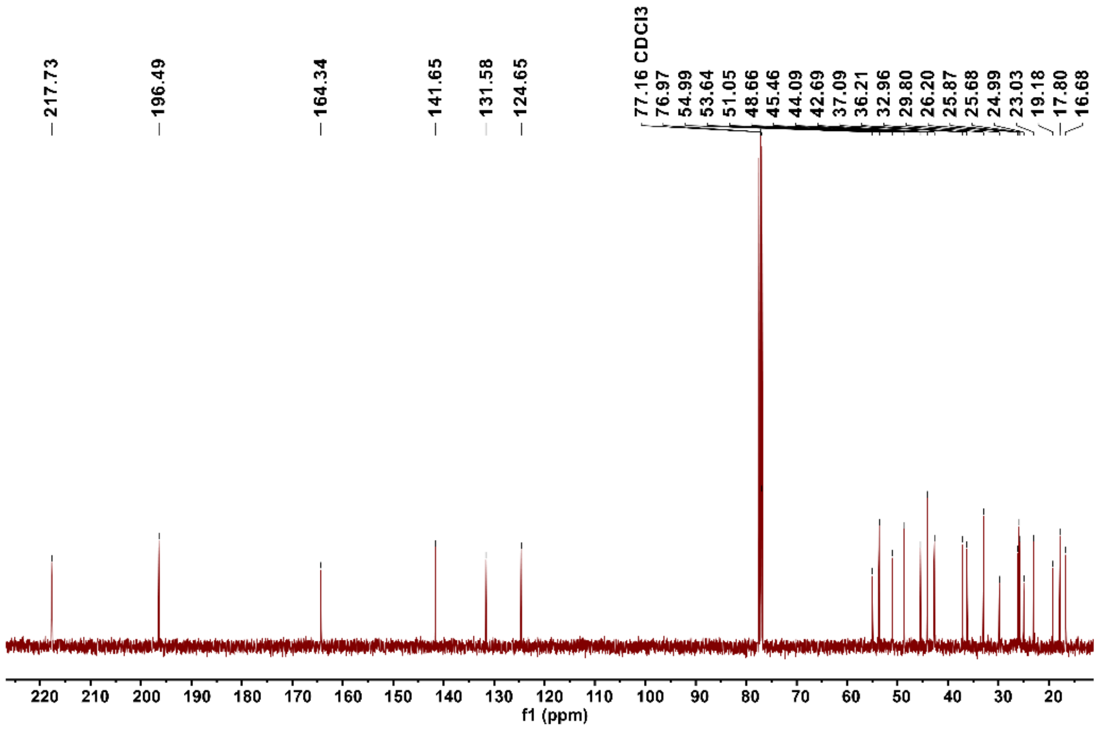

Figure S15 ^{13}C NMR spectrum of compound **3** (100 MHz, $CDCl_3$)

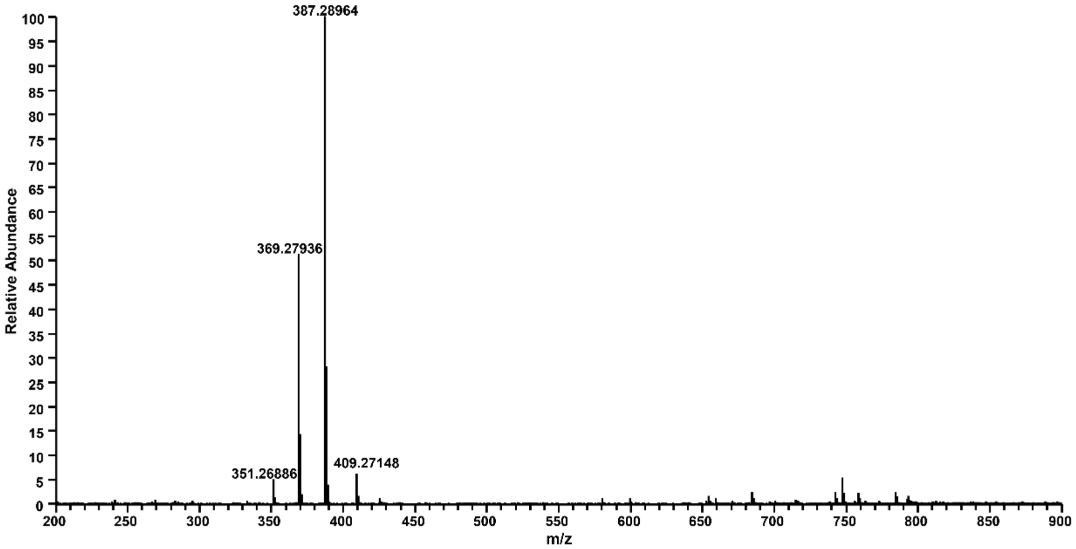

Figure S16 ESI-HRMS spetrum of compound **3**

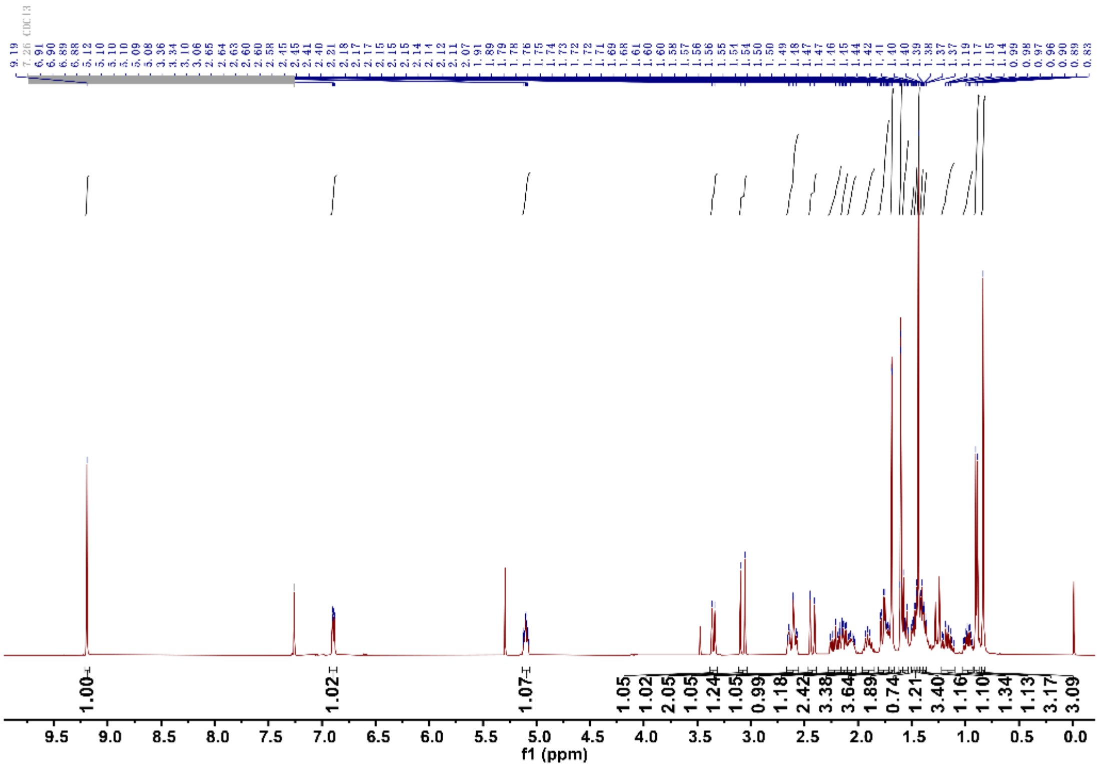

Figure S17 ^{1}H NMR spectrum of compound **4** (400 MHz, $CDCl_3$)

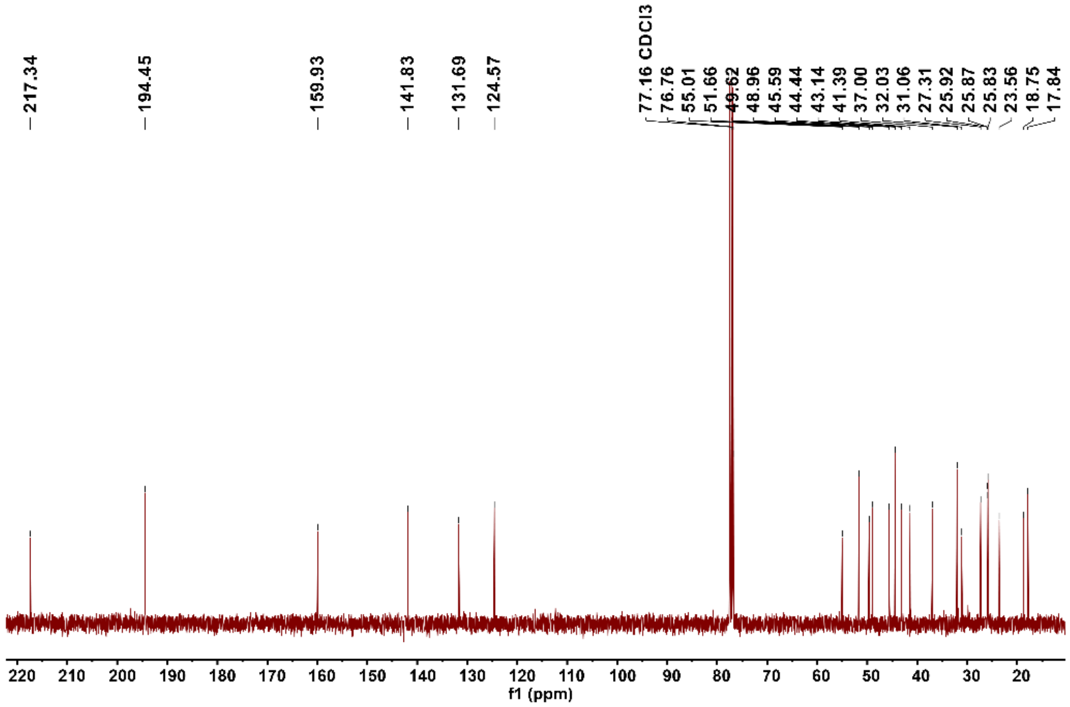

Figure S18 ^{13}C NMR spectrum of compound **4** (100 MHz, $CDCl_3$)

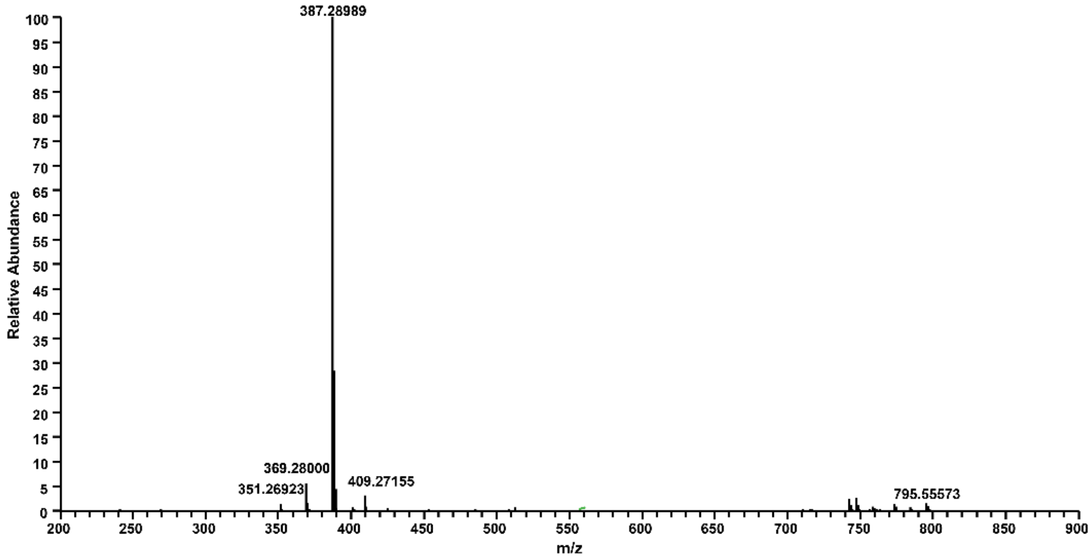

Figure S19 ESI-HRMS spetrum of compound **4**

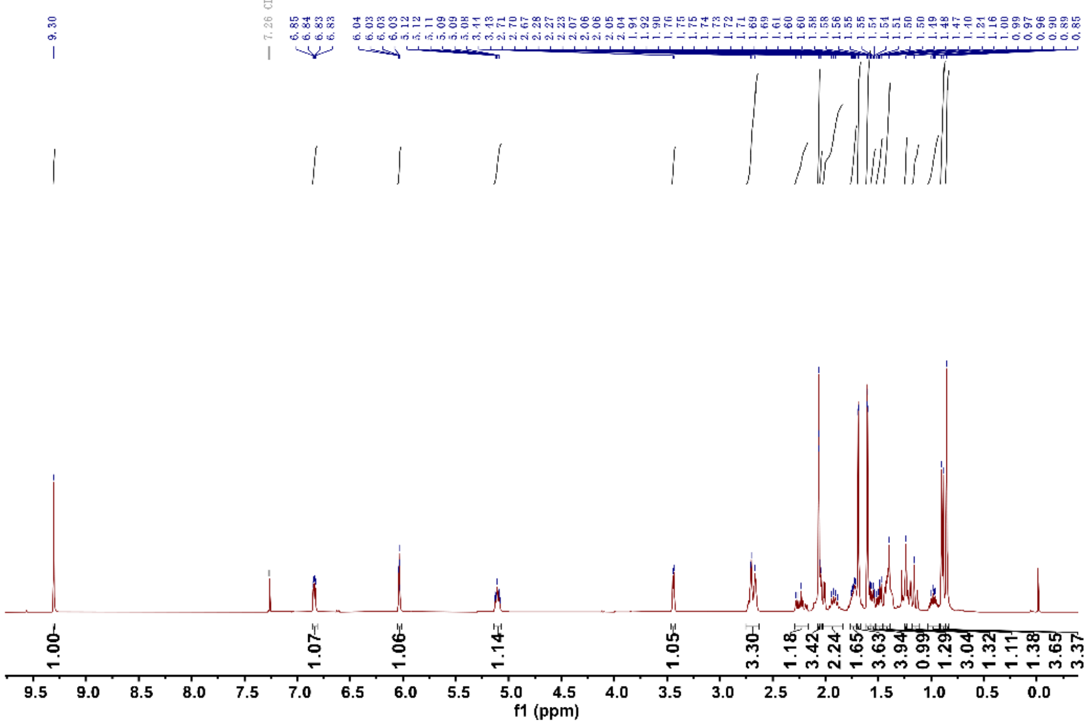

Figure S20 ^{1}H NMR spectrum of compound **5** (400 MHz, $CDCl_3$)

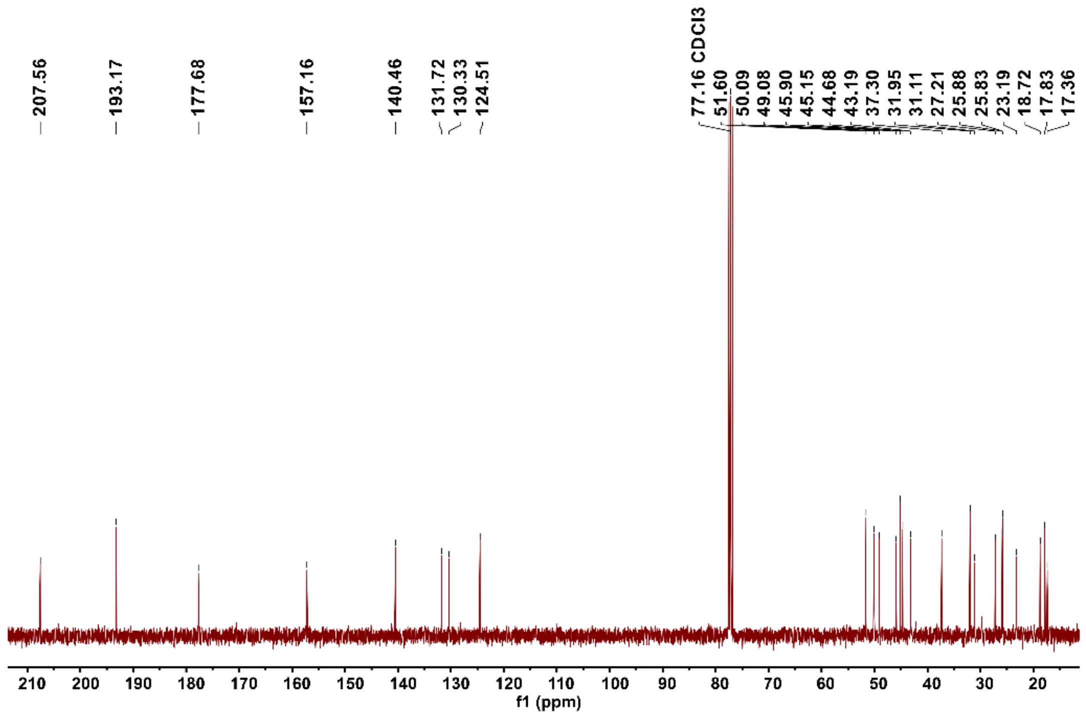

Figure S21 ^{13}C NMR spectrum of compound **5** (100 MHz, $CDCl_3$)

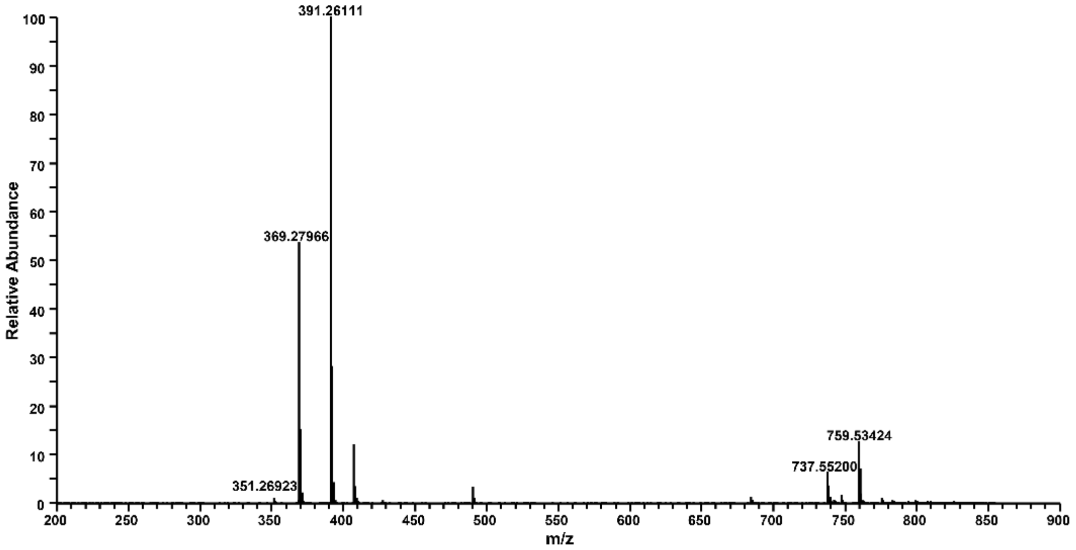

Figure S22 ESI-HRMS spetrum of compound **5**

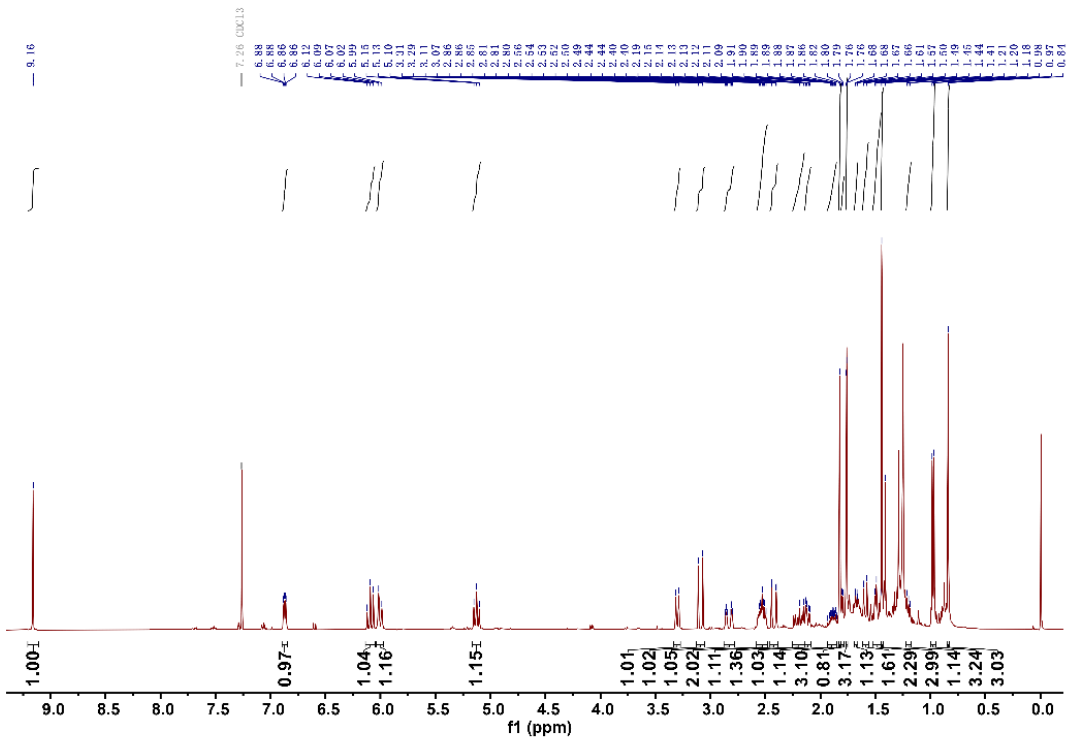

Figure S23 ^{1}H NMR spectrum of compound **6** (400 MHz, $CDCl_3$)

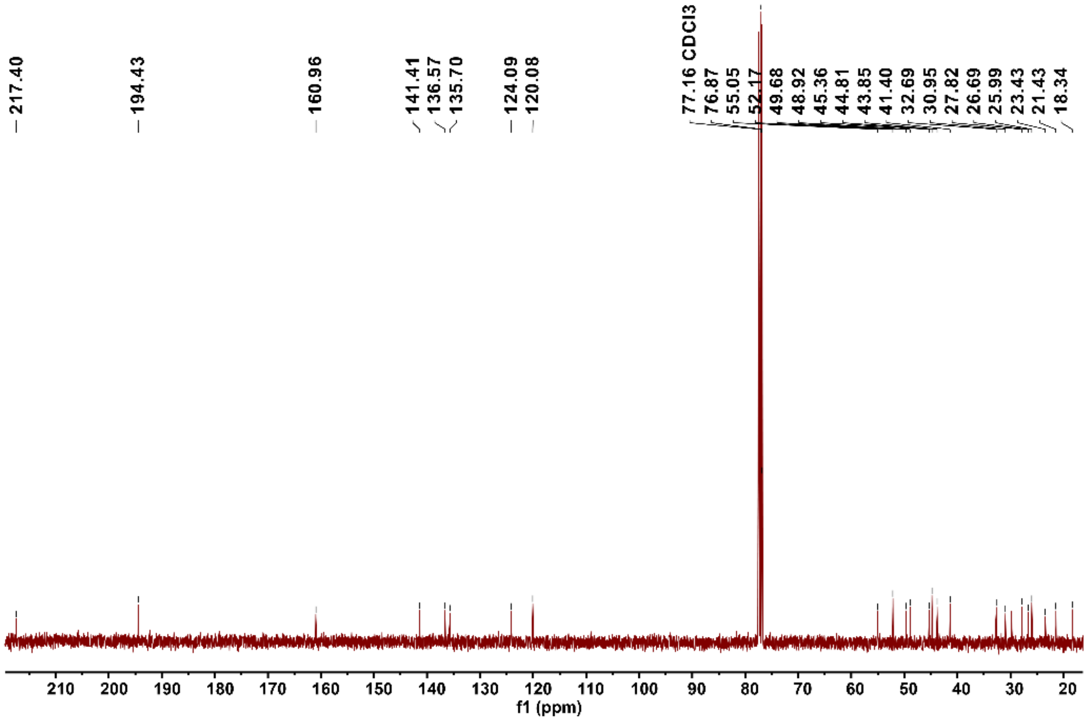

Figure S24 ^{13}C NMR spectrum of compound **6** (100 MHz, $CDCl_3$)

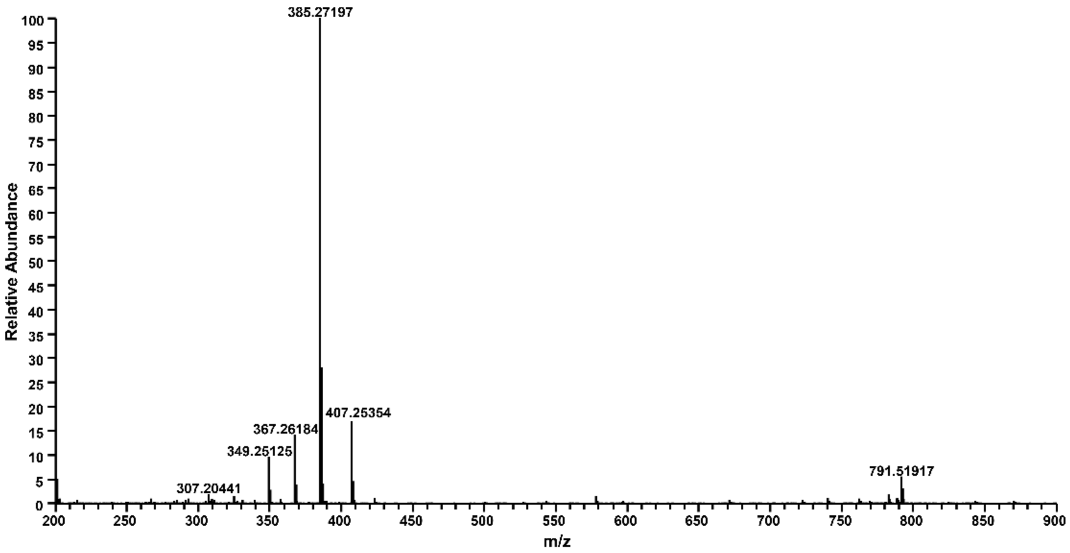

Figure S25 ESI-HRMS spetrum of compound **6**

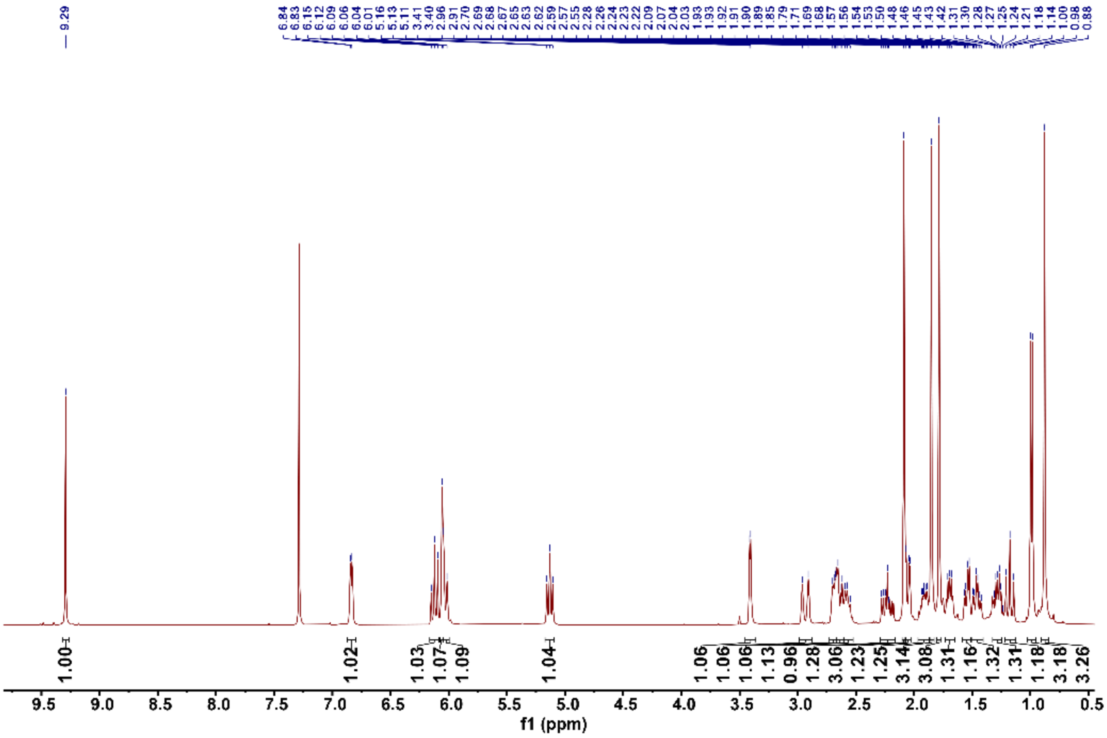

Figure S26 ^{1}H NMR spectrum of compound **7** (400 MHz, $CDCl_3$)

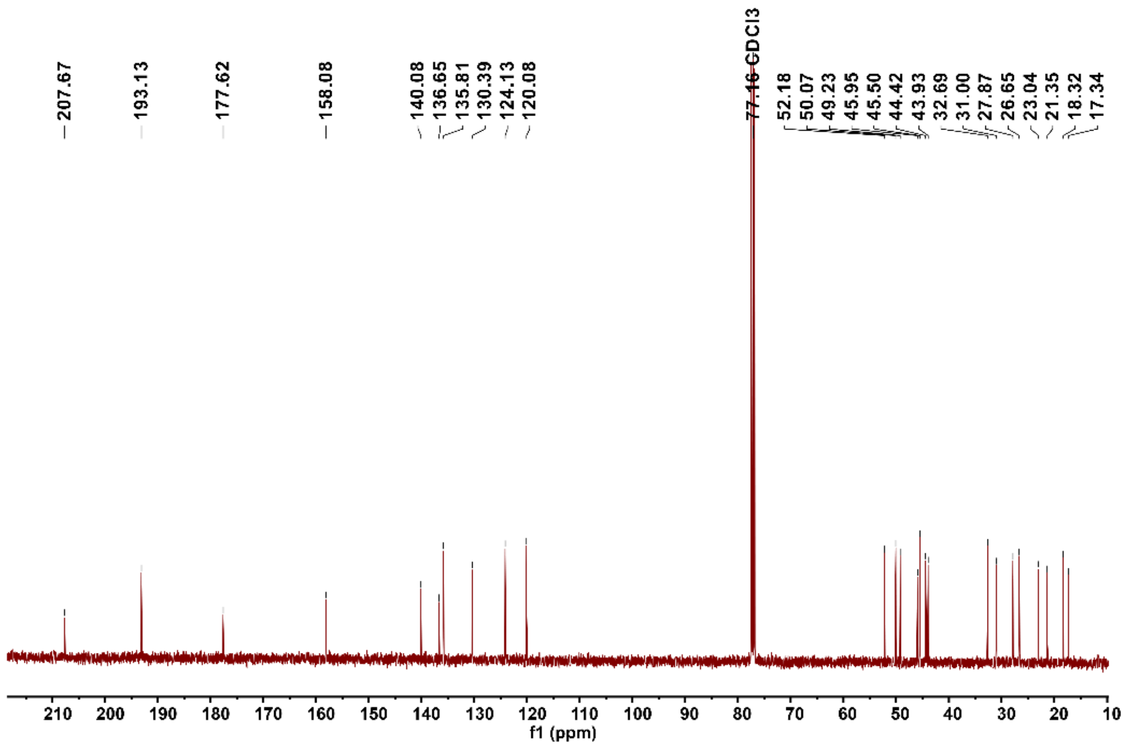

Figure S27 ^{13}C NMR spectrum of compound **7** (100 MHz, $CDCl_3$)

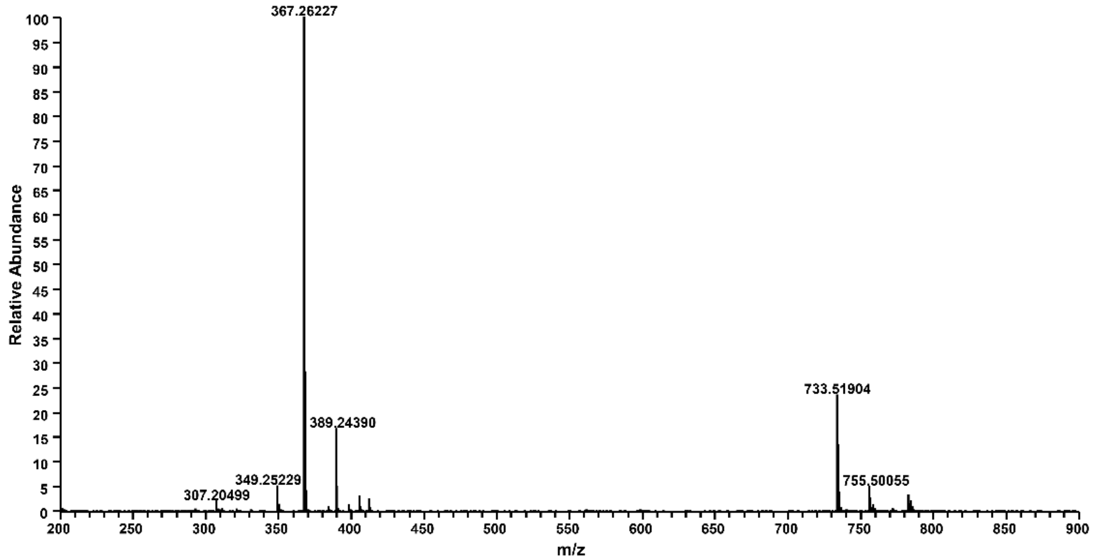

Figure S28 ESI-HRMS spetrum of compound **7**

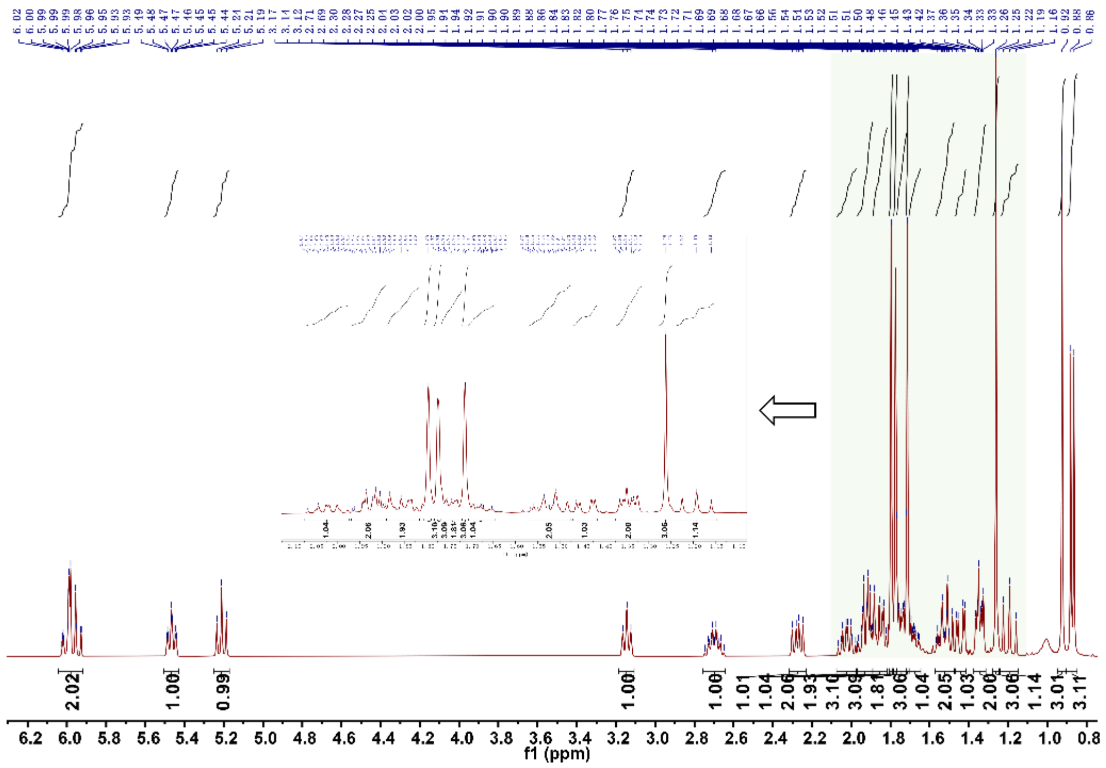

Figure S29 ^{1}H NMR spectrum of compound **10** (400 MHz, $CDCl_3$)

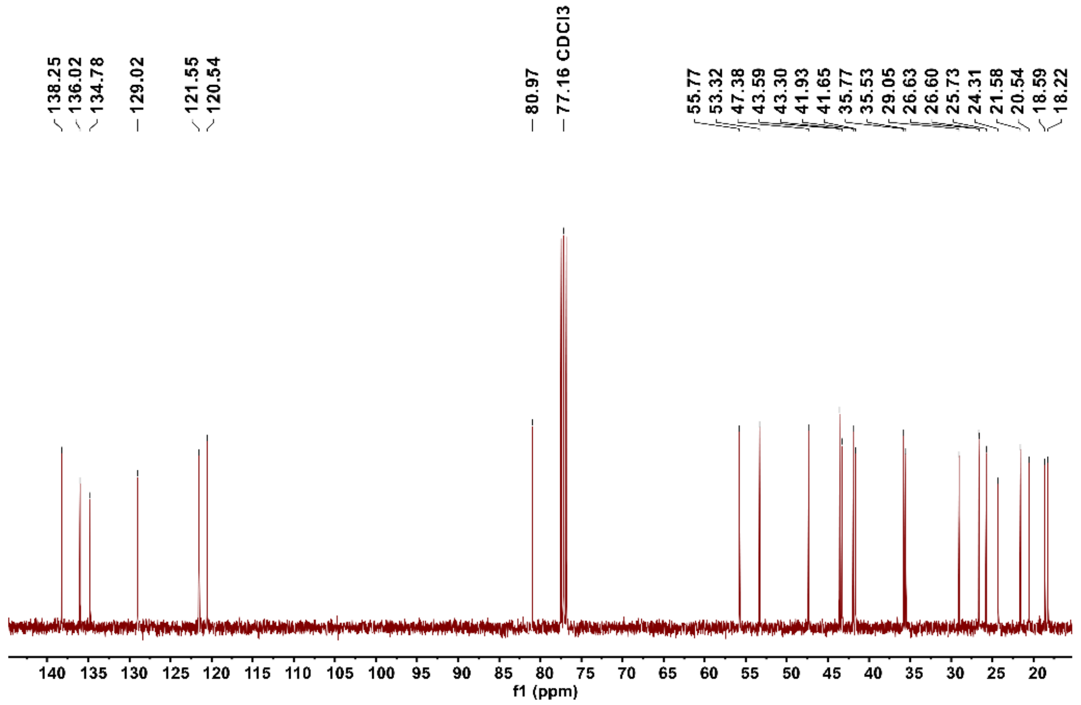

Figure S30 ^{13}C NMR spectrum of compound **10** (100 MHz, $CDCl_3$)

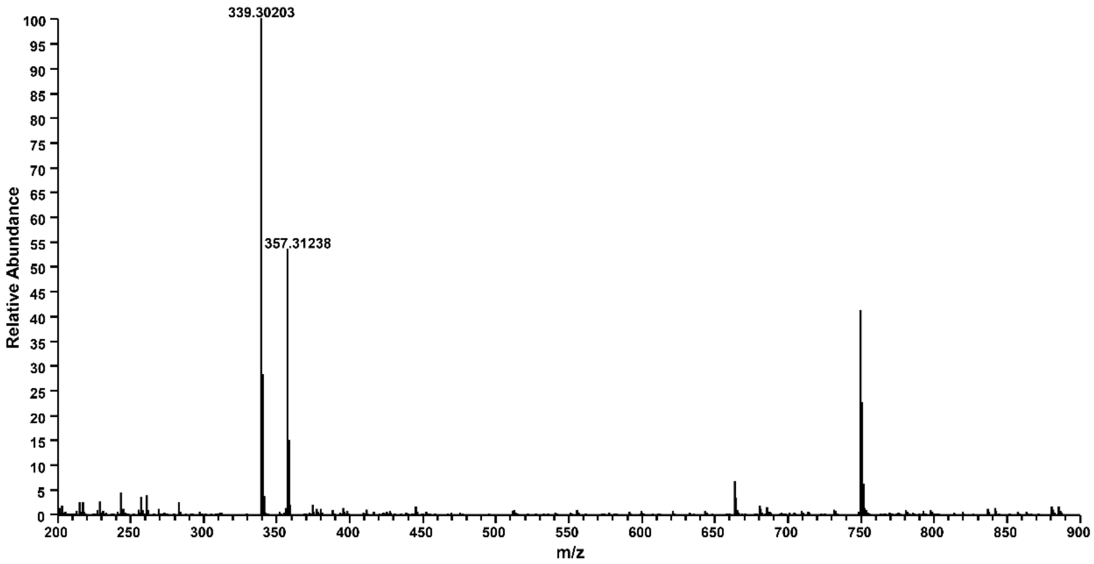

Figure S31 ESI-HRMS spetrum of compound **10**

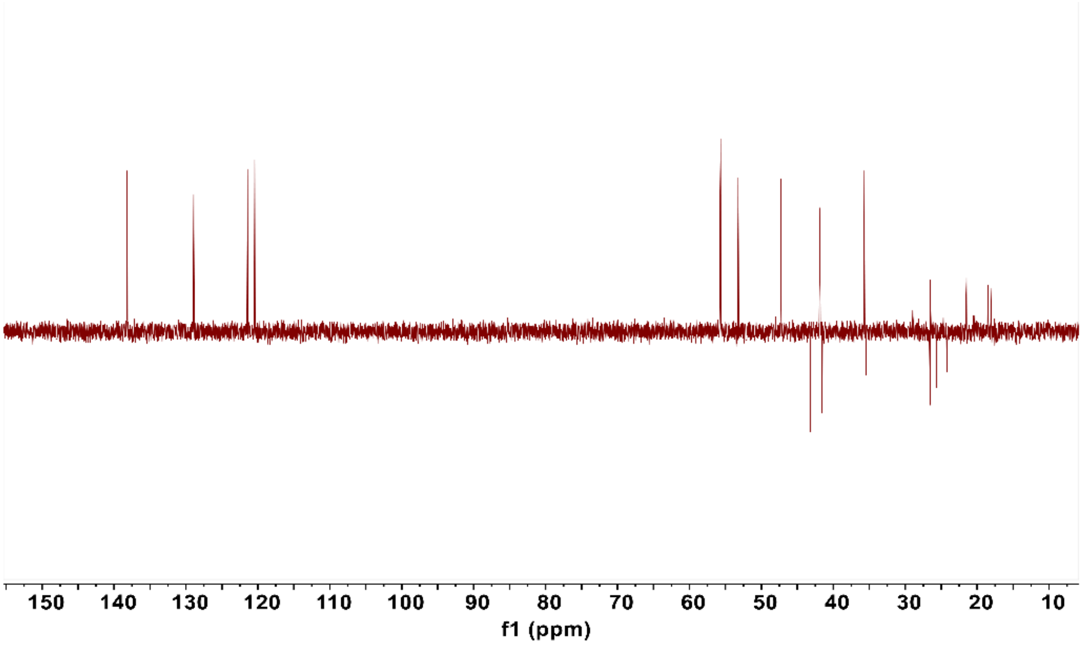

Figure S32 DEPT135 of compound **10** (100 MHz, $CDCl_3$)

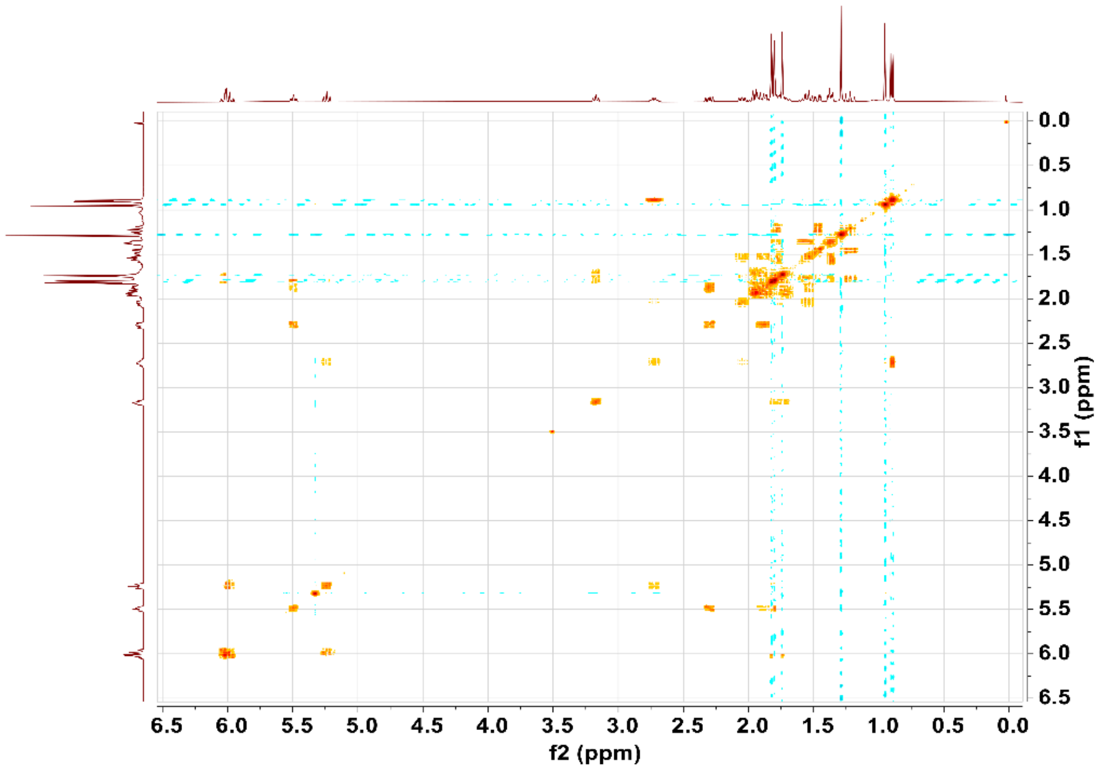

Figure S33 ^1H-^1H COSY spectrum of compound **10** (400 MHz, $CDCl_3$)

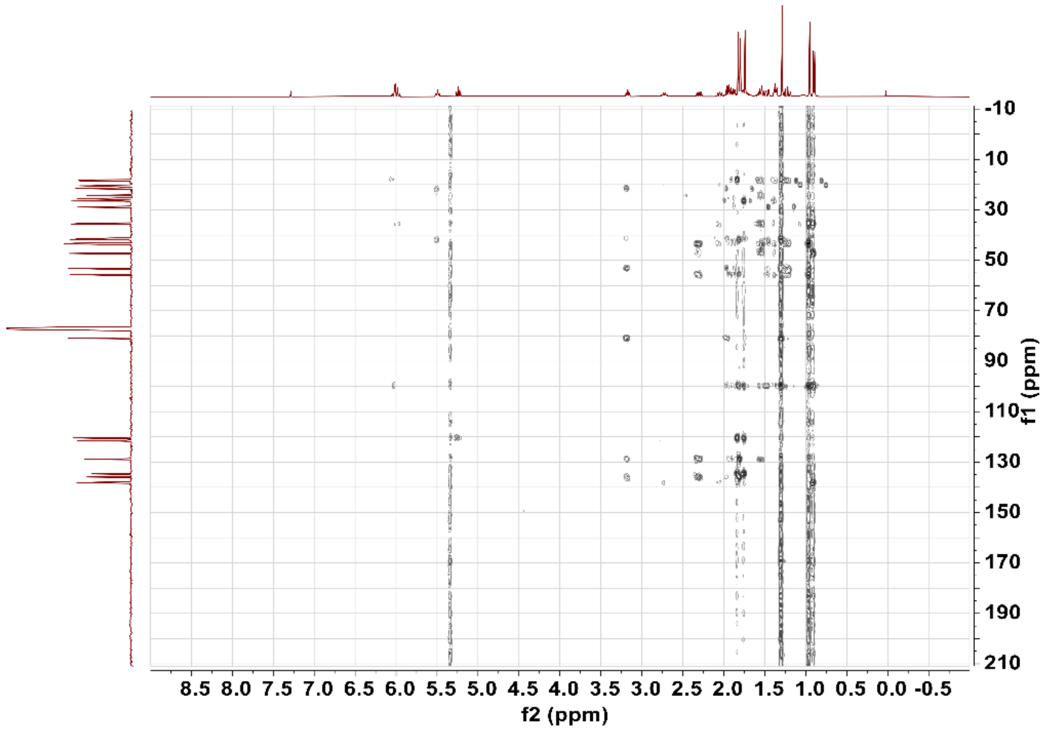

Figure S34 HMBC spectrum of compound **10** (400 MHz, $CDCl_3$)

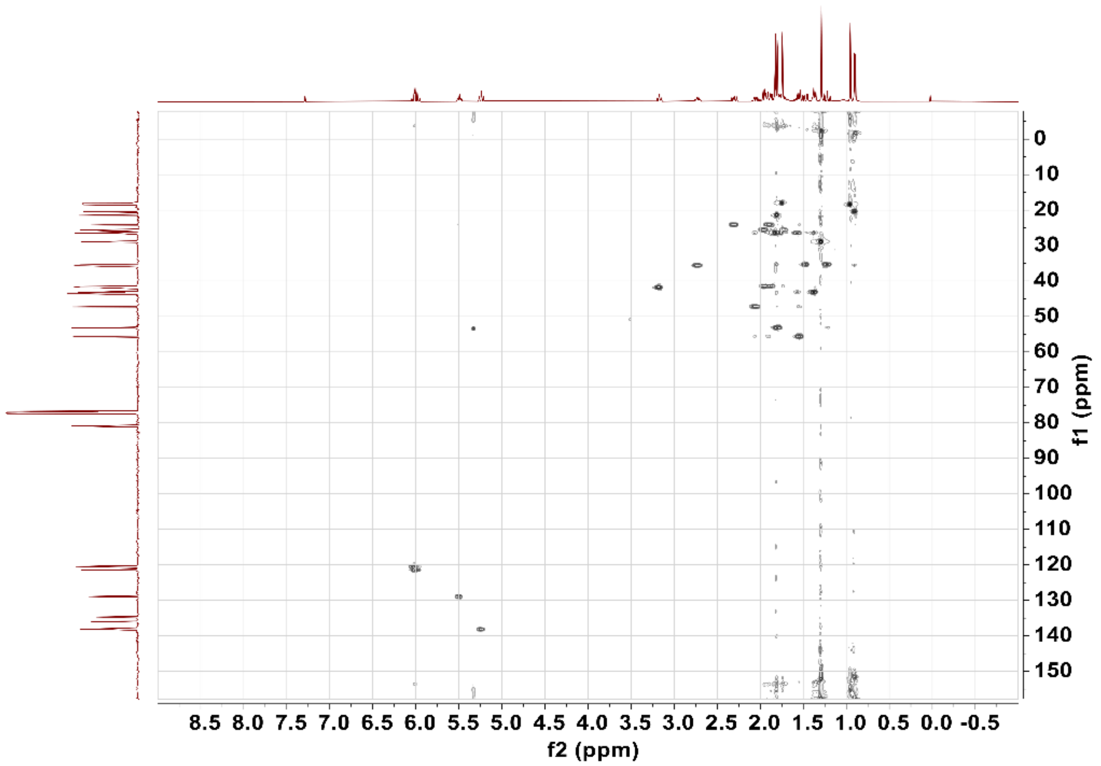

Figure S35 HSQC spectrum of compound **10** (400 MHz, $CDCl_3$)

Supplementary references

1. Wei, H., Itoh, T., Kinoshita, M., Nakai, Y., Kurotaki, M., & Kobayashi, M. Cytotoxic sesterterpenes, 6-epi-ophiobolin G and 6-epi-ophiobolin N, from marine derived fungus *Emericella variecolor* GF10. *Tetrahedron.* **2004,** 60, (28), 6015-6019.
2. Chiba, R., Minami, A., Gomi, K., & Oikawa, H. Identification of ophiobolin F synthase by a genome mining approach: a sesterterpene synthase from *Aspergillus clavatus*. *Org. Lett.* **2012,** 15, (3), 594-597.
3. Zhu, T., Lu, Z., Fan, J., Wang, L., Zhu, G., Wang, Y., Li, X., Hong, K., Piyachaturawat, P., Chairoungdua, A., & Zhu, W. Ophiobolins from the mangrove fungus *Aspergillus ustus*. *J. Nat. Prod.* **2018,** 81, (1), 2-9.
4. Chai, H., Yin, R., Liu, Y., Meng, H., Zhou, X., Zhou, G., Bi, X., Yang, X., Zhu, T., Zhu, W., Deng, Z., & Hong, K. Sesterterpene ophiobolin biosynthesis involving multiple gene clusters in *Aspergillus ustus*. *Sci. Rep.* **2016,** 6, 27181-27191.
5. Narita, K., Chiba, R., Minami, A., Kodama, M., Fujii, I., Gomi, K., & Oikawa, H. Multiple oxidative modifications in the ophiobolin biosynthesis: P450 oxidations found in genome mining. *Org. Lett.* **2016,** 18, (9), 1980-1983.
